# Achieving High Levels of NMR‐Hyperpolarization in Aqueous Media With Minimal Catalyst Contamination Using SABRE

**DOI:** 10.1002/chem.201702716

**Published:** 2017-07-19

**Authors:** Wissam Iali, Alexandra M. Olaru, Gary G. R. Green, Simon B. Duckett

**Affiliations:** ^1^ Department of Chemistry University of York Heslington, York YO10 5DD UK; ^2^ York Neuroimaging Centre The Biocentre, York Science Park Innovation Way Heslington, York YO10 5DD UK

**Keywords:** hyperpolarization, NMR spectroscopy, *para*-hydrogen, SABRE

## Abstract

Signal amplification by reversible exchange (SABRE) is shown to allow access to strongly enhanced ^1^H NMR signals in a range of substrates in aqueous media. To achieve this outcome, phase‐transfer catalysis is exploited, which leads to less than 1.5×10^−6^ mol dm^−3^ of the iridium catalyst in the aqueous phase. These observations reflect a compelling route to produce a saline‐based hyperpolarized bolus in just a few seconds for subsequent in vivo MRI monitoring. The new process has been called catalyst separated hyperpolarization through signal amplification by reversible exchange or CASH‐SABRE. We illustrate this method for the substrates pyrazine, 5‐methylpyrimidine, 4,6‐*d*_2_‐methyl nicotinate, 4,6‐*d*_2_‐nicotinamide and pyridazine achieving ^1^H signal gains of approximately 790‐, 340‐, 3000‐, 260‐ and 380‐fold per proton at 9.4 T at the time point at which phase separation is complete.

NMR is commonly used across a large number of disciplines, including chemistry and medicine, but is inherently insensitive because it probes a population difference between states that are close in energy. This population difference can be increased by employing hyperpolarization techniques, such as optical pumping, DNP^1^ or the use of *para*hydrogen (*p*‐H_2_),^2^ by *p*‐H_2_ induced polarization (PHIP),^3^ to increase sensitivity.

A form of PHIP, known as signal amplification by reversible exchange (SABRE),^4^ is used here to hyperpolarize a substrate in just a few seconds. One of the main advantages of SABRE is that it achieves this result without the incorporation of *p*‐H_2_ into the substrate. This technique utilizes a suitable catalyst^5^ to reversibly bind both H_2_ (*p*‐H_2_) and the substrate to assemble a species that can transfer spin order at low magnetic fields from *p*‐H_2_ into the substrate.^6^

One important objective of hyperpolarization lies in the area of magnetic resonance imaging (MRI) for use in medical diagnosis.^7^ In fact, employing hyperpolarized agents^8^ in applications such as tumor or metabolic‐flux imaging, is beginning to become a reality.^9^ The toxicity of the SABRE catalyst, solvent and substrate need to be minimized, however, before the SABRE method could be used clinically.

Currently, the best reported catalyst for SABRE is [IrCl(COD)(IMes)] (**1**),^10^ delivering ^1^H‐signal enhancements reaching about 50 % polarization in [D_4_]MeOH solutions, in which both catalyst and *p*‐H_2_ solubility is very high.^11^ Although previous studies have shown that less toxic [D_6_]ethanol/D_2_O mixtures can be employed, the level of signal gain is typically reduced.^12^ However, activity is seen in neat D_2_O even when catalyst activation can be slow in this solvent.^13^ Feiters et al., and Shi et al., respectively, prepared a water‐soluble catalyst for use with SABRE but the resulting enhancements in water were again weak when compared to those in methanol,^13^, ^14^ as were those achieved by heterogeneous catalysis.^15^ Here, we demonstrate how the principles of phase‐transfer catalysis can be used to improve the SABRE response in water while simultaneously achieving catalyst separation (Scheme 1). A related approach has been used very successfully with PHIP such that 10 % ^13^C‐polarization was achieved.^16^

**Scheme 1:**
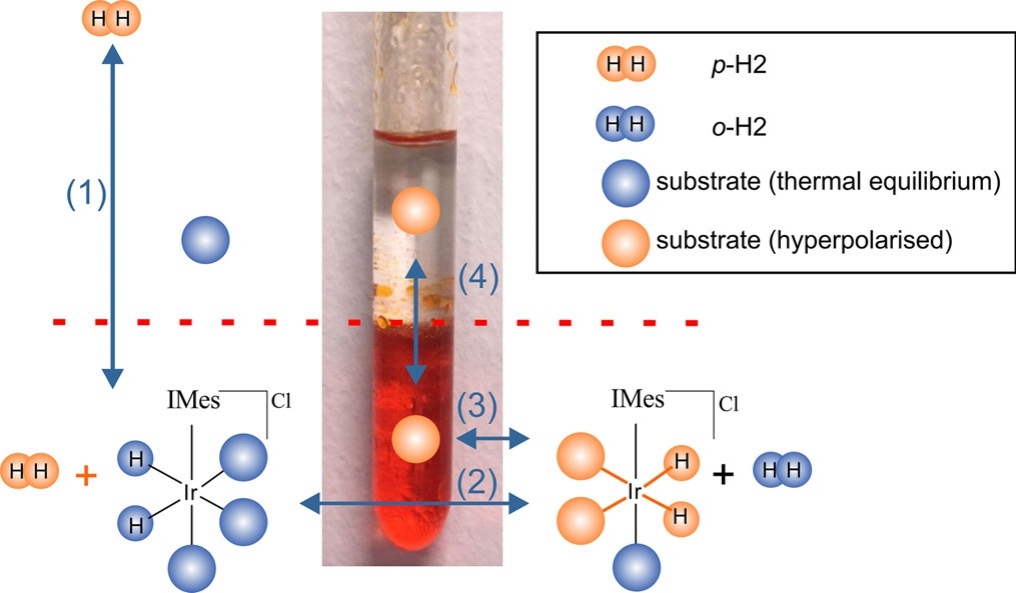
Partitioning of the SABRE catalyst and hyperpolarization target between the two immiscible phases of chloroform and water allows the principles of phase‐transfer catalysis to be employed in conjunction with *p*‐H_2_ to produce high levels of hyperpolarization in the aqueous phase with essentially no catalyst contamination.

To develop this method, a sample was prepared by combining 0.3 mL of a CDCl_3_ solution that contained 5 mm of **1** with 0.3 mL of a D_2_O solution containing 20 mm of the hyperpolarization target pyrazine (**pz**). As the organic and aqueous phases are immiscible, the lower CDCl_3_ layer retained the original orange colour due to the catalyst, whereas the water remained colourless. When H_2_ was added on top of the solution, and the sample shaken to dissolve it, a rapid reaction ensued that led to the CDCl_3_ phase becoming deeply red in colour due to the formation of [Ir(H)_2_(IMes)(**pz**)_3_]Cl. Shaking, however, causes the two initially distinct phases to emulsify prior to separating over 60 seconds. Once the aqueous phase is separated, it remains colourless; thus, these changes are readily discernible optically, as shown in Scheme 1. We note that chloroform is partially soluble in water, ultimately reaching a 0.5 % level by volume,^17^ although we have assessed it reaching a 0.08 % level here 10 seconds after mixing. Given the toxicity of chloroform,^18^ an N_2_ purge would be needed to lower this level in the aqueous phase if it were to be used clinically because the environmental protection agencies recommended water quality criteria specify a limit of 0.07 mg L^−1^.^19^

These changes can be readily assessed by acquiring a series of 1D projections of the samples ^2^H‐NMR signal along the *z*‐axis of the tube using well‐established gradient echo methods (Figure 1). When the corresponding ^1^H image is recorded to track the weaker **pz** signal of this sample, the slow separation of the two phases can be assessed, and ultimately a 62.5:37.5 **pz** partitioning in favour of the CDCl_3_ phase is seen.

**Figure 1:**
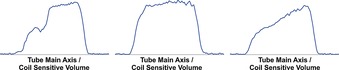
^2^H signal intensity profiles derived from the solvent response as a function of distance from the bottom of the NMR tube (right). (a) Prior to shaking the separated 0.3 mL of a CDCl_3_ containing 5 mm of **1** and the 0.3 mL of D_2_O containing 20 mm **pz**, (b) immediately after shaking an emulsion with no formal phase separation evident and (c) 25 seconds later when partial phase separation is evident.

When this biphasic mixture is exposed to a 3 bar pressure of *p*‐H_2_, and the sample shaken in the stray field of the magnet for 10 seconds, SABRE occurs, as shown by a high resolution ^1^H NMR spectrum recorded immediately after the sample was inserted in the high‐field spectrometer. The resulting hyperpolarized **pz** response shows a 645±15 fold signal enhancement (2 % polarization) per proton when compared to that recorded under Boltzmann conditions for a phase separation time of zero. We probed the enhanced **pz** response in a series of 1D ^1^H‐signal intensity projections along the *z*‐axis of the tube as a function of increasing phase‐separation time. These results reveal that initially the **pz** signal intensity is slightly weighted towards the lower end of the tube in which the aqueous phase dominates (Figure 2).

**Figure 2:**
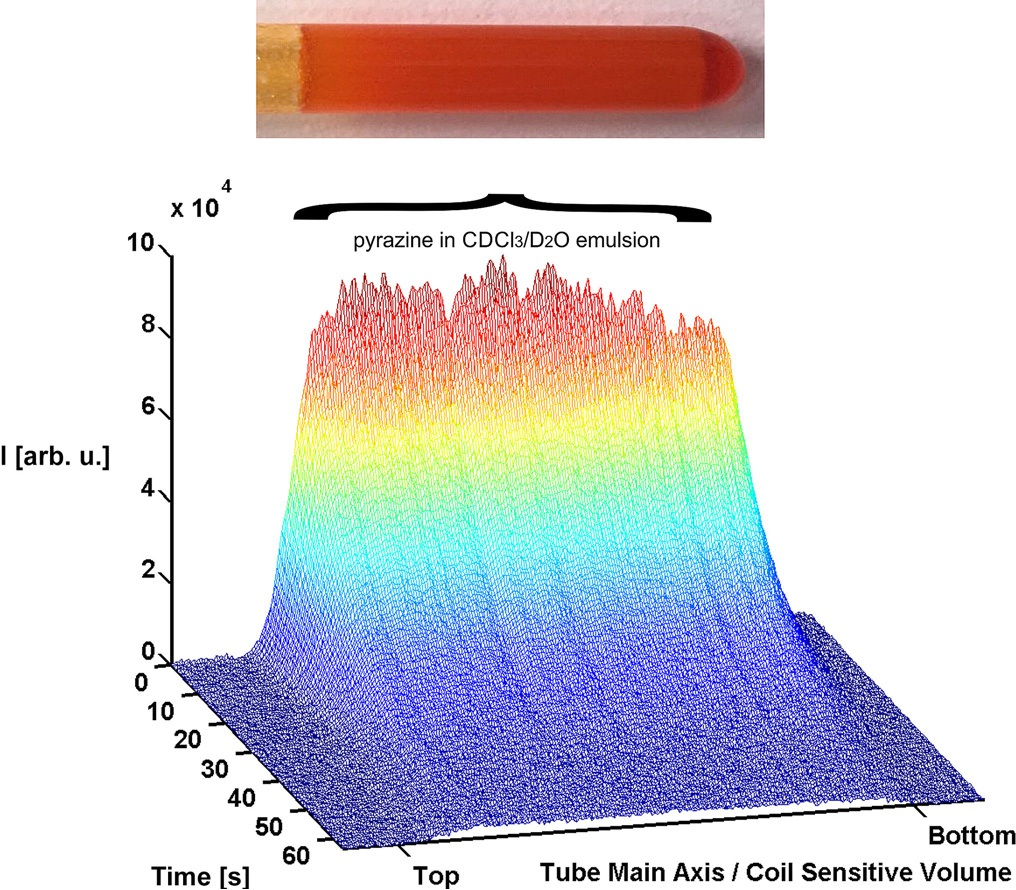
^1^H NMR signal intensity as a function of distance from the bottom of the NMR tube that results after emulsion‐based SABRE‐derived phase‐transfer catalysis to produce a hyperpolarized **pz** response. The fall in **pz** signal intensity as a function of time (s), reveals that the hyperpolarized response decays before phase‐separation is achieved.

The level of this response reduces in size with increasing separation time due to relaxation and we note that complete phase separation is not seen before relaxation destroys the hyperpolarized response in such a sample. As the aim of this work was to achieve **pz** hyperpolarization in water without catalyst contamination, rapid phase separation is essential. A further series of test samples were therefore prepared to explore the effect of varying the amount of CDCl_3_ and D_2_O, while keeping the total sample volume constant at 0.6 mL. The level of signal gain proved to increase by 25±7 % on moving from pure CDCl_3_ to a 17 % loading, but again, full relaxation occurs before the phase separation is complete.

As saline represents an ideal solvent for in vivo applications, we repeated these studies using 0.35 mL of D_2_O doped with 0.16 % *w*/*v* of NaCl and 0.25 mL of CDCl_3_. The effect of this change was dramatic with the resulting signal gain increasing to 790±20 fold (2.5 % polarization) per proton after 10 seconds when phase separation is achieved. These results are illustrated in Figure 3, with the hyperpolarized **pz** signal area in the organic and aqueous phases having a ratio of 48.8:51.2 after 10 seconds. The retained **pz** signal gain after 15 seconds is 400 fold (1.3 % polarization), and when the fully relaxed image was recorded, the ratio of **pz** in the two phases is about 23:77 respectively, which shows that the salt is beneficial in improving the aqueous **pz** loading under these conditions. We used UV monitoring to compare the rate of transfer of **pz** from H_2_O into CHCl_3_ in the presence (0.16 % *w*/*v*) and absence of NaCl and observed an approximate 4‐fold increase, which means the reverse process is also accelerated. Furthermore, the red colour associated with the catalyst is still selectively retained in the organic phase. This statement was supported by the fact that UV spectroscopy on the aqueous phase revealed that its concentration was less than 1.5×10^−6^ mol dm^−3^ after 10 seconds, in agreement with the failure to see a SABRE response when this layer was tested. Hence, we conclude that we have established a rapid route to produce a hyperpolarized bolus in D_2_O that is essentially catalyst‐free in which adding salt plays a beneficial role.^20^ We note that CDCl_3_ contaminates the aqueous phase at a 0.08 % level (5 mg L^−1^), which is reduced to 0.06 % by NaCl at this point.

**Figure 3:**
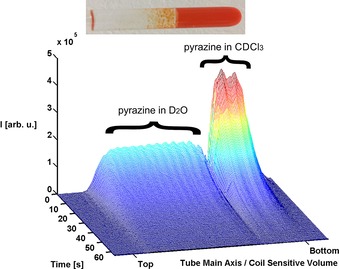
^1^H NMR signal intensity as a function of distance from the bottom of the NMR tube that results after SABRE‐derived phase‐transfer catalysis in the presence of NaCl. In this case, phase‐separation occurs significantly faster than the relaxation of the hyperpolarized **pz** response.

To show that it is possible to exploit this behaviour in the collection of MRI data, we replaced the 5 mm NMR tube with a 10 mm sample and used a triple axis gradient system to acquire 2D one‐shot images of slices parallel to the main axis of the tube (Figure 4). We also measured single voxel spectra (SVS) of **pz** in CDCl_3_ and D_2_O, which confirm the origin of these signals as the hyperpolarized agent, distributed between the organic and aqueous phases (see the Supporting Information). The hyperpolarized responses presented in Figure 4 were recorded 15 seconds after completion of the initial hyperpolarization step.

**Figure 4:**
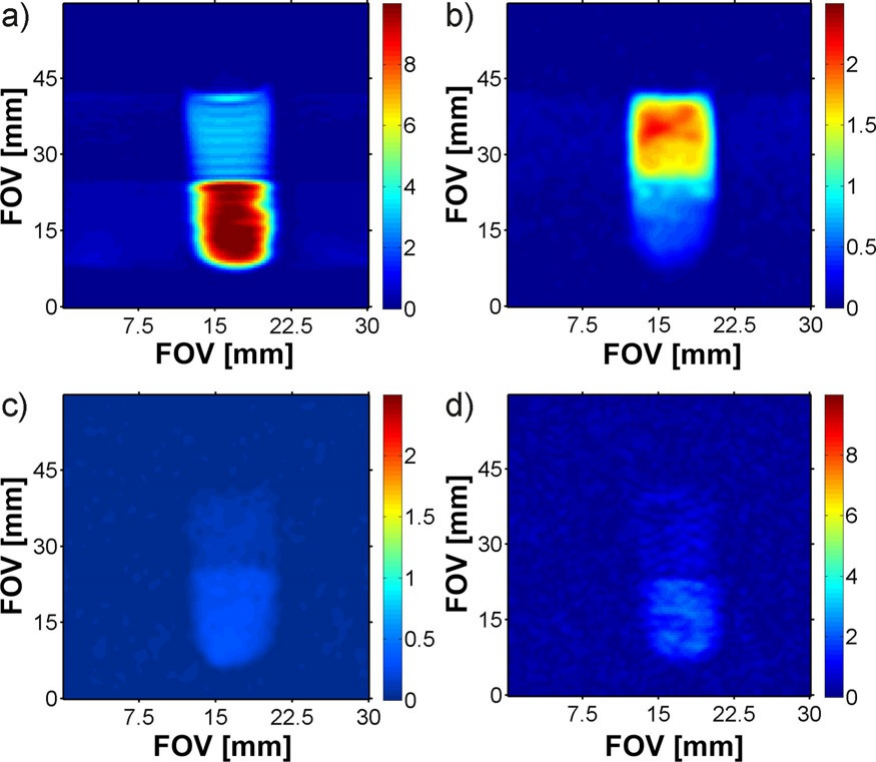
2D‐^1^H‐MRI images of slices parallel to the *B*_o_ field encoding **pz** (100 mm) and pyridazine (50 mm) responses under hyperpolarized (a and b, respectively, 15 seconds after mixing) and thermal conditions (c and d, respectively). Partitioning between the separated aqueous (upper) and organic (lower) phases is clearly visible with the pyridazine response being particularly notable.

As a result of recently published results that showed a high pH dependence to SABRE efficiency,^21^ we also tested the effects of adding the salts, that is, NaCO_2_Me, NH_4_CO_2_Me, NH_4_Cl, NaOH, NaH(CO_3_) and Na_2_CO_3_. These results are described in the Supporting Information and reveal that under spectroscopic examination, separate signals for **pz** can be seen in the two distinct phases in the majority of cases. This point confirms a role for phase transfer without the need for imaging. NaCl, however, proved to deliver the best separation times and enhancement levels.

Considering recent ^15^N‐NMR developments,^22^ we have also demonstrated that when the high‐resolution ^13^C and ^15^N responses of **pz** at a 100 mm concentration are examined, strong signals are detected in the aqueous and chloroform phases at different frequencies (Figure 5, 180‐ and 3000‐fold enhancements, respectively). Furthermore, because there is a wide interest in diversifying the range of agents hyperpolarized by SABRE,^23^ we then tested the generality of this approach by reference to the substrates 5‐methylpyrimidine, 4,6‐*d*_2_‐methyl nicotinate, 4,6‐*d*_2_‐nicotinamide and pyridazine. All four agents produced SABRE‐enhanced resonances in the aqueous phase, coupled with phase separation times of less than 10 seconds and good catalyst separation (signal gains of approximately 340, 3000, 260 and 380‐fold, respectively under similar conditions to those used for **pz** earlier, see the Supporting Information).The scale of the 4,6 *d*_2_‐methyl nicotinate response is particularly noteworthy, and will reflect its long relaxation time,^11^ whereas the pyridazine MRI data of Figure 4 c and d illustrate how the partitioning between the phases varies with agent, in this case leading to a very strong aqueous signal.

**Figure 5:**
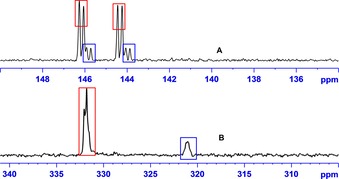
**(A**) SABRE ^13^C response of **pz** in the water (blue) and chloroform (orange) phases after transfer at 30 G. (B) SABRE ^15^N NMR **pz** response in the water (blue) and chloroform (orange) phases after transfer at about 0 G in a μ‐metal shield.

Hence, we believe that this new and simple catalyst separated hyperpolarization through signal amplification by reversible exchange (CASH‐SABRE) approach reflects an exciting route to produce high levels of hyperpolarization in a biocompatible aqueous medium with very limited catalyst contamination. We have demonstrated here that ^1^H, ^13^C and ^15^N detection is possible and are now seeking to develop this approach further through the introduction of chloroform‐optimized catalysts and new substrates, while simultaneously exploring new solvent combinations to further minimize contamination of the aqueous phase.

## Conflict of interest

The authors declare no conflict of interest.

## Supporting information

As a service to our authors and readers, this journal provides supporting information supplied by the authors. Such materials are peer reviewed and may be re‐organized for online delivery, but are not copy‐edited or typeset. Technical support issues arising from supporting information (other than missing files) should be addressed to the authors.

Supplementary
