# Supplementary material for: Achieving High Levels of NMR‐Hyperpolarization in Aqueous Media With Minimal Catalyst Contamination Using SABRE
Source: Chemistry. 2017 Jul 19;23(44):10491–5. doi: 10.1002/chem.201702716 (PMC5582620; doi:10.1002/chem.201702716)
Supplement: Supplementary file 1 — Supplementary [file CHEM-23-10491-s001.pdf]

CHEMISTRY
A European Journal

Supporting Information

Achieving High Levels of NMR-Hyperpolarization in Aqueous Media With Minimal Catalyst Contamination Using SABRE

Wissam Iali,[a] Alexandra M. Olaru,[a] Gary G. R. Green,[b] and Simon B. Duckett*[a]

chem_201702716_sm_miscellaneous_information.pdf

Supporting Information
©Wiley-VCH 2016
69451 Weinheim, Germany

Achieving High Levels of NMR-Hyperpolarization in Aqueous Media With Minimal Catalyst Contamination via SABRE

W. Iali,[a] A. M. Olaru,[a] G. G. R. Green,[a] and S. B. Duckett*[a]

Abstract: Signal amplification by reversible exchange (SABRE) is shown to allow access to strongly enhanced ^{1}H NMR signals in a range of substrates in aqueous media. In order to achieve this outcome phase-transfer-catalysis is exploited which leads to less than less than 1.5 x 10^{-6} mol dm^{-3} of the iridium catalyst in the aqueous phase. These observations reflect a compelling route to produce a saline based hyperpolarized bolus in just a few seconds for subsequent *in vivo* MRI monitoring. The new process has been called CAtalyst Separated Hyperpolarization via Signal Amplification By Reversible Exchange or CASH-SABRE. We illustrate this method for the substrates pyrazine, 5-methylpyrimidine, 4,6-d_2-methyl nicotinate, 4,6-d_2-nicotinamide and pyridazine achieving ^{1}H signals gains of approximately 790-, 340-, 3000-, 260- and 380-fold per proton at 9.4 T at the time point where phase separation is complete.

DOI: 10.1002/anie.2016XXXXX

[a] Dr. W. Iali, Dr. A. M. Olaru, Prof. G. G. R. Green, Prof. S. B. Duckett*
Centre for Hyperpolarisation in Magnetic Resonance, Department of Chemistry
University of York
Innovation Way, Heslington, York, UK, YO10 5NY
E-mail: simon.duckett@york.ac.uk

Supporting information for this article is given via a link at the end of the document.

WILEY-VCH

Table of Contents

Experimental Procedures

Materials 3
SABRE analysis 3
1D MRI experiments 3
2D MRI experiments 4
Single Voxel Spectroscopy Experiments 4

Results and Discussion

1. NMR spectroscopy results

1.1 Optimization experiments performed on pyrazine dissolved in $CDCl_3/D_2O$ mixtures 5
1.2 SABRE hyperpolarization of pyrazine in $CDCl_3/D_2O$ mixtures in the presence of NaCl 5
1.3 SABRE hyperpolarization of pyrazine in $CDCl_3/D_2O$ mixtures in the presence of other salts 8

2. 1D MRI results

2.1 SABRE hyperpolarization of pyrazine in $CDCl_3/D_2O$ mixtures 13
2.2 SABRE hyperpolarization of pyrazine in $CDCl_3/D_2O$ mixtures in the presence of NaCl 13
2.3 SABRE hyperpolarization of pyrazine in $CDCl_3/D_2O$ mixtures in the presence of other salts 17
2.4 SABRE hyperpolarization of other substrates in $CDCl_3/D_2O$ mixtures in the presence of NaCl 21
2.5 SABRE hyperpolarization of pyrazine in $CDCl_3/D_2O$ mixtures in the presence of NaCl: effect of temperature 26

3. 2D MRI results

3.1 2D MRI of SABRE hyperpolarized pyrazine in $CDCl_3/D_2O$ mixtures in the presence of NaCl 27
3.2 2D MRI of SABRE hyperpolarized pyrazine in $CDCl_3/D_2O$ mixtures in the presence of other salts 30
3.3 2D MRI of SABRE hyperpolarized pyridazine in $CDCl_3/D_2O$ mixtures in the presence of NaCl 31

4. Quality control by UV-Vis and transport between the phases 32

WILEY-VCH

Experimental Procedures

Materials

All of the experimental procedures associated with this work were carried out under nitrogen using standard Schlenk techniques. The solvents used were dried using an Innovative Technology anhydrous solvent system, or distilled from an appropriate drying agent under nitrogen. The catalyst precursor [Ir(IMes)(COD)Cl] (**1**) employed in this work was synthesized by established procedures according to literature methods.[1] Deuterated chloroform ($CDCl_3$), deuterated water (D_2O), deuterated ethanol (EtOD), pyrazine (**2**), 5-methylpyrimidine, and pyridazine were purchased from Sigma Aldrich and used as supplied. 4,6-d_2-methyl nicotinate and 4,6-d_2-nicotinamide were synthesized as described by Rayner *et al*.[1] The level of chloroform contamination in the aqueous phase was assessed by signal comparison to a known internal DMSO reference.

SABRE analysis

All samples have been prepared in standard 5 mm NMR tubes equipped with Young taps. In a typical experiment set, arrays of NMR measurements were collected using either 4 equivalents of substrate (1-fold excess) to 5 mM of iridium or 20 equivalents of substrate (17-fold excess) to 5 mM iridium dissolved in mixtures of $CDCl_3$ and D_2O. The exact composition of each sample analyzed is specified detailed in Table S1.

Table S1. Detailed composition of samples studied in this work.

Sample ID	Ligand concentration [mmol]	$CDCl_3$ volume [µl]	D_2O volume [µl]	Additive	Additive Concentration [mmol]
**Group 1**					
**1a**	0.012	600	0	-	-
**1b**	0.012	500	100	-	-
**1c**	0.012	400	200	-	-
**1d**	0.012	300	300	-	-
**1e**	0.012	200	400	-	-
**1f**	0.012	100	500	-	-
**1g**	0.012	20	580	-	-
**Group 2**					
**2a**	0.012	600	0	-	
**2b**	0.012	400	200	NaCl	0.0170
**2c**	0.012	300	300	NaCl	0.0170
**2d**	0.012	200	400	NaCl	0.0170
**2e**	0.012	100	500	NaCl	0.0170
**2f**	0.012	250	350	NaCl	0.0085
**2g**	0.060	300	300	NaCl	0.0170
**2h**	0.060	300	300	NaCl	0.0340
**2i**	0.060	300	300	NaCl<br>EtOD, 50 µl	0.0340
**Group 3**					
**3a**	0.012	300	300	NaOH	0.0085
**3b**	0.012	300	300	Na_2CO_3	0.0085
**3c**	0.012	300	300	$NaHCO_3$	0.0085
**3d**	0.012	300	300	$CH_3COO^-Na^+$	0.0085
**3e**	0.012	300	300	$CH_3COO^-NH_4^+$	0.0085
**3f**	0.012	300	300	$NH4^+Cl^-$	0.0085
**3g**	0.060	300	300	$NH_4^+Cl^-$<br>NaCl	0.0085<br>0.0170
**3h**	0.030	300	300	NaCl	0.0500

After adding p-H_2 at 3 bar pressure, ^1H NMR spectra were recorded using π/2 excitation pulses after shaking the sample in a magnetic field between 0 and 65 G. Enhancement factors were calculated by using the ratio of the integral areas of individual resonances in the hyperpolarized NMR spectrum and the corresponding spectrum collected under normal H_2 and Boltzmann equilibrium conditions respectively.

Similar experiments have been performed in order to assess the possibility to polarize heteronuclei such as ^{13}C and ^{15}N. ^{13}C hyperpolarization experiments have been performed using polarization transfer fields between 0 and 65 G, while ^{15}N data has been acquired at 0 G field, by inserting the tube and shaking the sample in a µ-metal shield designed to shield the sample form the environmental magnetic field.

The ^{13}C and ^{15}N NMR signal enhancements were calcualted relative to an external standard using the following formula:

$$E = \frac{S_{pol} \times N_{pol}}{S_{ref\ unpol} \times N_{ref\ unpol}}$$

E = Signal enhancement
S_{pol} = Integral of polarized samples signal
$S_{ref\ unpol}$ = Intergral of unpolarized reference (^{15}N,^{13}C labelled urea of known concentration)

N_{pol} = Effective concentration of ^{13}C or ^{15}N in polarized sample
$N_{ref\ unpol}$ = Effective concentration of ^{13}C or ^{15}N in unpolarized reference

1D MRI experiments

All 1D MRI experiments have been performed using a 400 MHz Bruker Avance spectrometer equipped with a *z*-gradient of maximum strength of 0.536 T/m. As a function of the observed nucleus, the experiments can be separated in two categories:

- 1D projections of the ^{2}H signal amplitude on the *z* direction (parallel to the magnet bore and the NMR sample tube). These experiments have been performed with the purpose of observing the spatial distribution of the two solvents.

- 1D projections of the ^{1}H signal amplitude on the *z* direction (parallel to the magnet bore and the NMR sample tube). In a typical experiment, 128 projections have been acquired immediately after the shaking process. These experiments have been performed with the purpose of observing the spatial distribution of the substrate in thermal equilibrium conditions, after shaking in the presence of *o*-H_2 and well as after shaking in the presence of *p*-H2.

2D MRI experiments

All 2D MRI experiments have been performed using a 400 MHz Bruker Avance spectrometer equipped with a micro imaging gradient set with maximum amplitude of 1T/m and a double resonance birdcage coil with a diameter of 30 mm. All samples have been prepared in 10 mm diameter standard NMR tubes. The hyperpolarization step has achieved by shaking the sample under *p*-H_2 for 10 seconds at 65G in the stray field of the magnet. Images have been acquired using the rapid acquisition schemes based on spin echoes (RARE) and gradient echoes respectively (Steady State Free Precession-SSFP). The ^{1}H MRI acquisition parameters were, as follows:

- For SSFP images: field of view 60 x 60 mm^2, slice thickness 2.5 mm, matrix size 64 x 64 (zero-filled to 128 x 128) leading to a nominal 2D resolution of 0.94 x 0.94 mm^2 (digital resolution 0.47 x 0.47 mm^2). TE/TR 2/4 ms. Repetition time between two consecutive image acquisition: 600 ms. Excitation pulse angle: 30°.

- For RARE images: field of view 30 x 60 mm^2, slice thickness 2.5 mm, matrix size 64 x 64 (zero-filled to 128 x 128) leading to a nominal 2D resolution of 0.47 x 0.94 mm^2 (digital resolution 0.23 x 0.47 mm^2). $TE/TE_{eff}/TR$: 4/4/1100 ms. Echo train length: 64.

A sine bell squared filter has been applied prior to the Fourier transform to minimize the contribution of noise in the images. Data post processing has been done using home developed routines in Prospa (Magritek) and MATLAB (MathWorks).

Single Voxel Spectroscopy (SVS) experiments

SVS experiments have been performed using the setup described in section 1.4. ^{1}H SABRE hyperpolarized spectra of pyrazine have been acquired by selecting two separate voxels (matrix size 5x5x5 mm^3) located parallel to the tube's vertical axis, as depicted in Figure S1. In order to minimize artefacts caused by diffusion and turbulence on the time scale of the experiment, an outer volume suppression scheme (OVS) has been used prior to data acquisition.

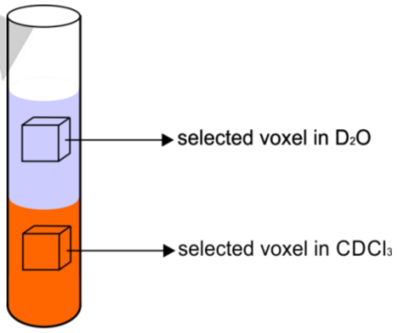

Figure S1. Schematic drawing of the NMR tube containing the sample and the position of the two voxels used for spectra acquisition.

Results and Discussion

1. NMR spectroscopy results

1.1 Optimization experiments performed on pyrazine dissolved in $CDCl_3/D_2O$ mixtures

In order to determine the optimal composition of the biphasic solvent that is to be used for SABRE catalysis, ^1H hyperpolarization experiments have been performed on a series of samples in which the ratio of the inorganic to organic phase has been varied. For each sample between five and ten hyperpolarization experiments were performed by shaking the sample in the stray field of the magnet at ~ 30 G for 10 seconds and immediately acquiring a ^1H spectrum after the hyperpolarization transfer step had been completed. The enhancement factor presented is the average value, taken from the integral areas of the hyperpolarized resonances divided by the corresponding area in a spectrum that was acquired under Boltzmann equilibrium conditions. The composition of each sample, together with the corresponding enhancement and associated errors, are presented in Table S2.

Table S2. Enhancements obtained for samples **1a**-**1g**, together with the associated experimental errors.

Sample	$CDCl_3$ Volume [μL]	D_2O Volume [μL]	Enhancement	Error [±]
1a	600	0	512	19
1b	500	100	564	18
1c	400	200	649	33
1d	300	300	645	16
1e	200	400	690	22
1f	100	500	650	24
1g	20	580	281	29

1.2 SABRE hyperpolarization of pyrazine in $CDCl_3/D_2O$ mixtures in the presence of NaCl.

Figures S2-S7 detail the effect of solvent composition on the level of SABRE hyperpolarization of pyrazine (see Table S1).

Sample 2a: 1-fold excess pyrazine; $CDCl_3$:D_2O ratio 0.6:0

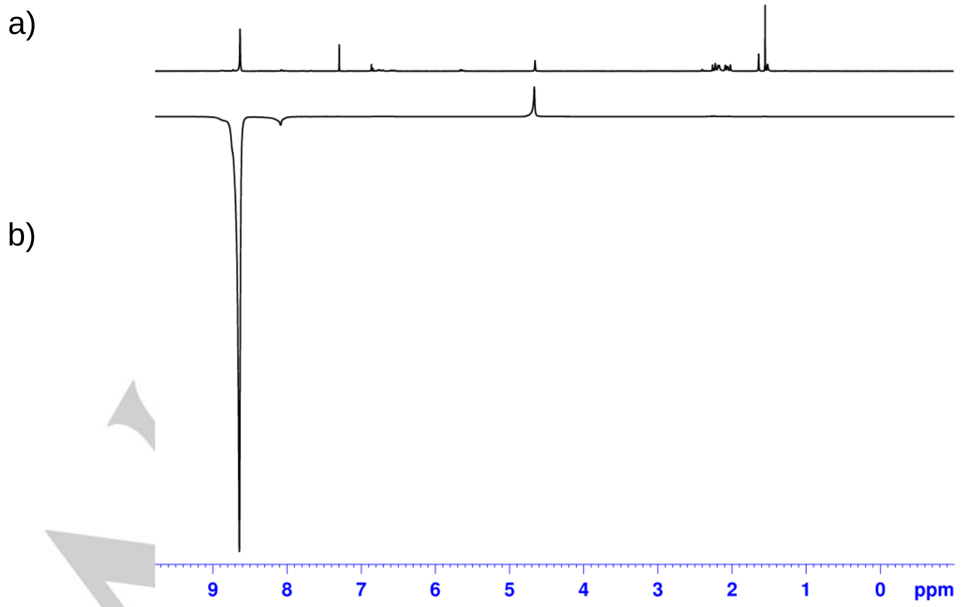

Figure S2. ^1H NMR spectra of sample **2a**. (**a**) Boltzmann equilibrium conditions (x16 vertical expansion relative to (b)), (**b**) SABRE hyperpolarized trace detailing 540-fold signal enhancement.

Sample 2b: 1-fold excess pyrazine; $CDCl_3$:D_2O ratio 0.4:0.2

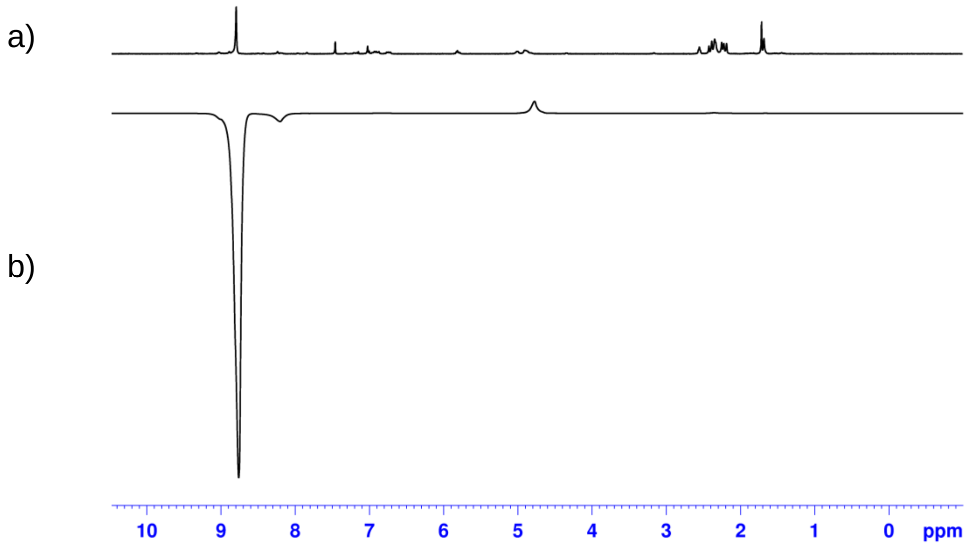

Figure S3. ^1H NMR spectra of sample **2b**. (a) Boltzmann equilibrium conditions (x16 vertical expansion relative to (b)), (b) SABRE hyperpolarized trace detailing 520-fold signal enhancement.

Sample 2c: 1-fold excess pyrazine; $CDCl_3$:D_2O ratio 0.3:0.3

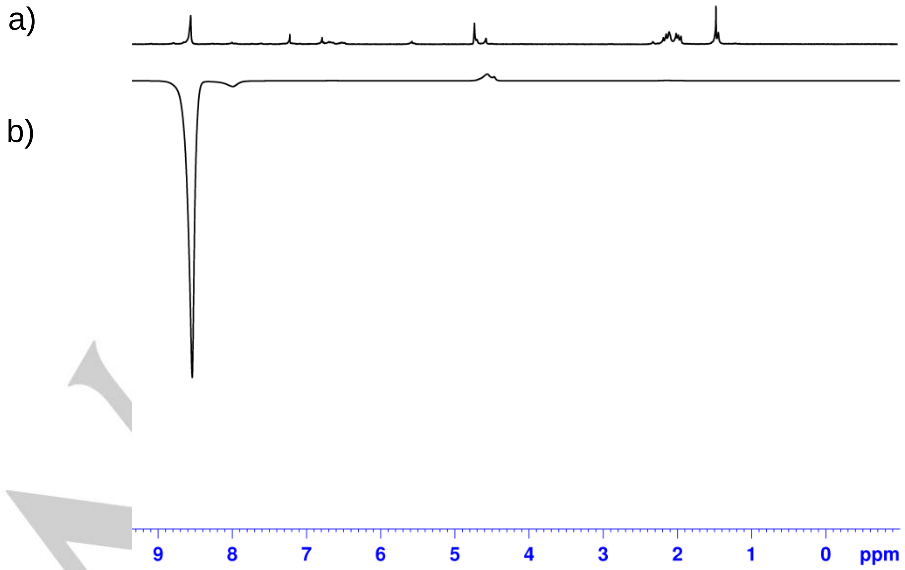

Figure S4. ^1H NMR spectra of sample **2c**. (a) Boltzmann equilibrium conditions (x8 vertical expansion relative to (b)), (b) SABRE hyperpolarized trace detailing 560-fold signal enhancement.

Sample 2d: 1-fold excess pyrazine; $CDCl_3$:D_2O ratio 0.2:0.4

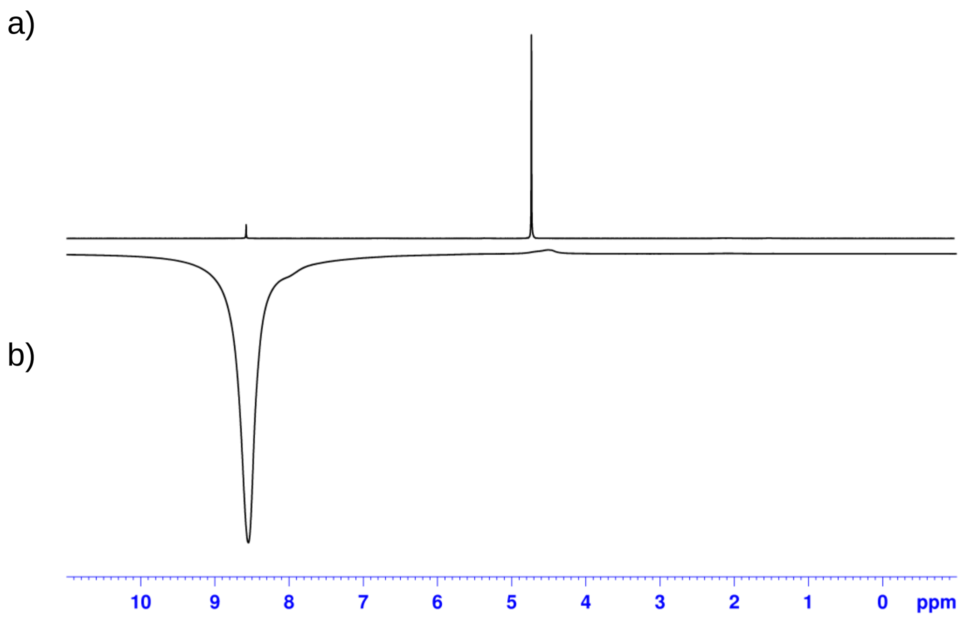

Figure S5. ^{1}H NMR spectra of sample **2d**. (a) Boltzmann equilibrium conditions (same vertical expansion as (b)), (b) SABRE hyperpolarized trace detailing 780-fold signal enhancement.

Sample 2e: 1-fold excess pyrazine; $CDCl_3$:D_2O ratio 0.1:0.5

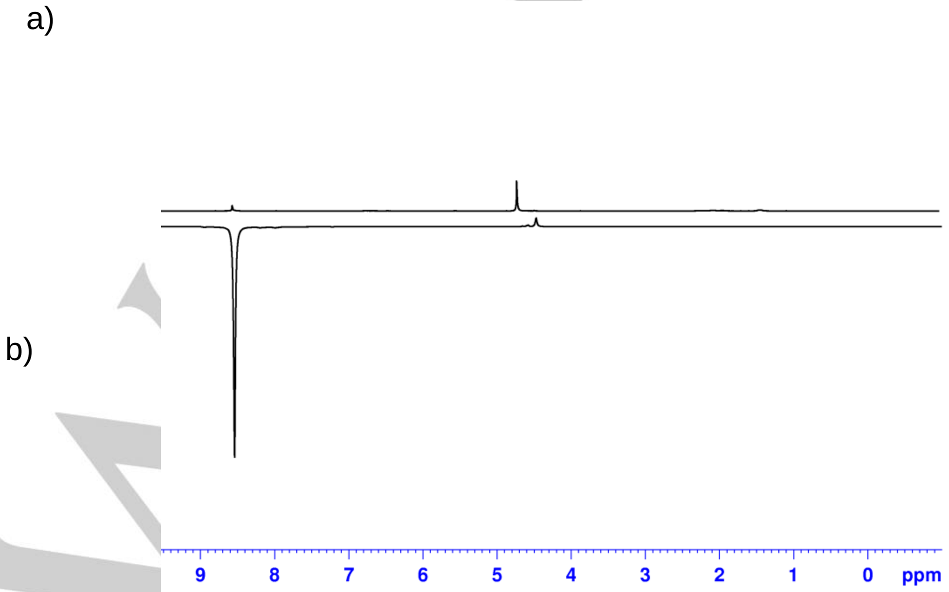

Figure S6. ^{1}H NMR spectra of sample **2e**. (a) Boltzmann equilibrium conditions (x16 vertical expansion relative to (b)), (b) SABRE hyperpolarized trace detailing 285-fold signal enhancement.

Sample 2f: 1-fold excess pyrazine; $CDCl_3$:D_2O ratio 0.25:0.35

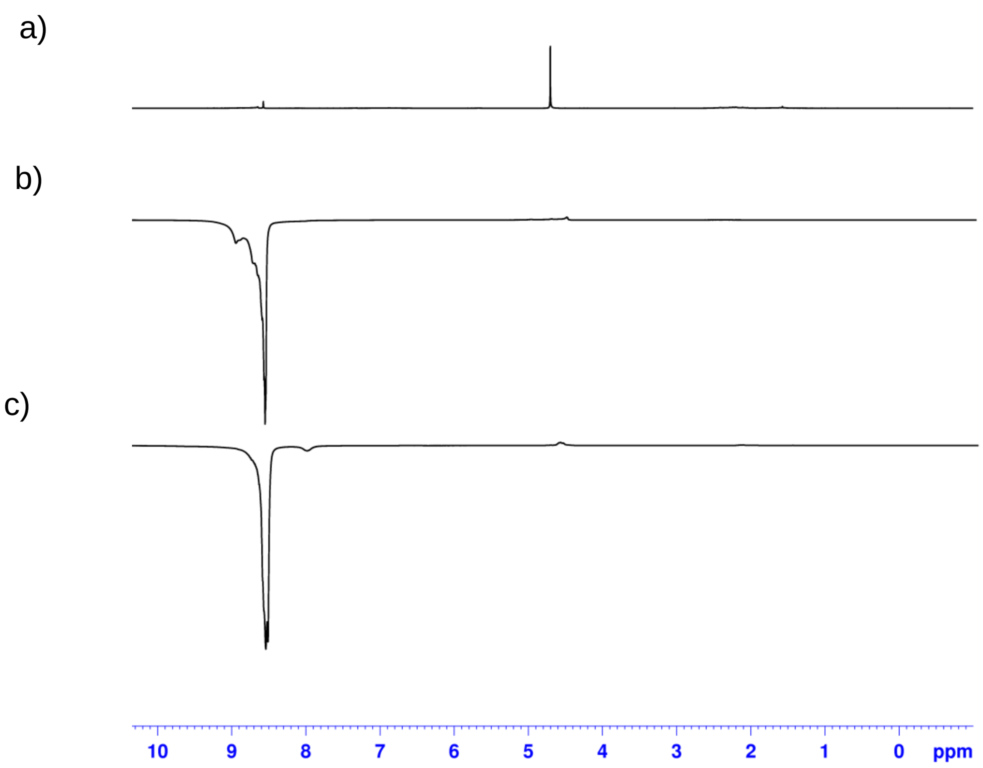

Figure S7. ^{1}H NMR spectra of sample **2f**. (a) Boltzmann equilibrium conditions (x8 vertical expansion relative to (b)), (b) SABRE hyperpolarized trace recorded 10 seconds after transfer and (**c**) SABRE hyperpolarized trace recorded 30 s after transfer.

1.3 SABRE hyperpolarization of pyrazine in $CDCl_3$/D_2O mixtures in the presence of other salts

Sample 3a: 1-fold excess pyrazine + NaOH

A sample was prepared using 1-fold excess of ligand relative to the active iridium catalyst (5 mM) in a mixture of 0.3 ml $CDCl_3$ and 0.3 D_2O. NaOH (0.0085 mmol) was then added as the separation promoting agent. When examining the result obtained after acquiring a 90° spectrum under Boltzmann equilibrium conditions, it can be seen that NaOH addition leads to a clear difference in the chemical shift of the pyrazine resonance being detected in the two different solvents.

The pyrazine ^{1}H NMR signal that was typically observed as a singlet in the previous experiments now appears now as a sharp, narrow peak (corresponding to the ligand dissolved in the water phase) and a low-intensity, broad resonance, shifted downfield from the former, corresponding to the substrate that is present in the organic phase (Figure S8).

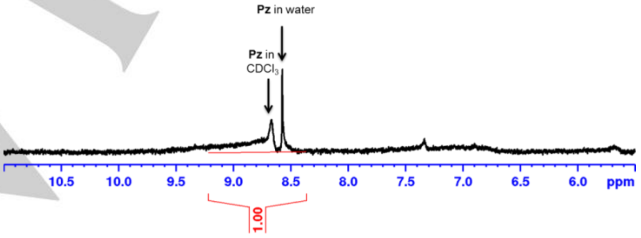

Figure S8. ^{1}H NMR spectrum of sample **3a** acquired under thermal equilibrium conditions (x8).

When examining the analogous spectra acquired after SABRE hyperpolarization, the chemical shift difference cannot be resolved if the measurement is performed immediately after the polarization transfer step, due to the extreme line broadening artefacts that are introduced by the fact that these data are acquired while the mixture separates and stabilizes (motion artefacts reduced). However, if

the mixture is allowed to separate prior to data acquisition, the chemical shift difference between the resonances in the distinct organic and inorganic phases can be clearly detected (Figure S9).

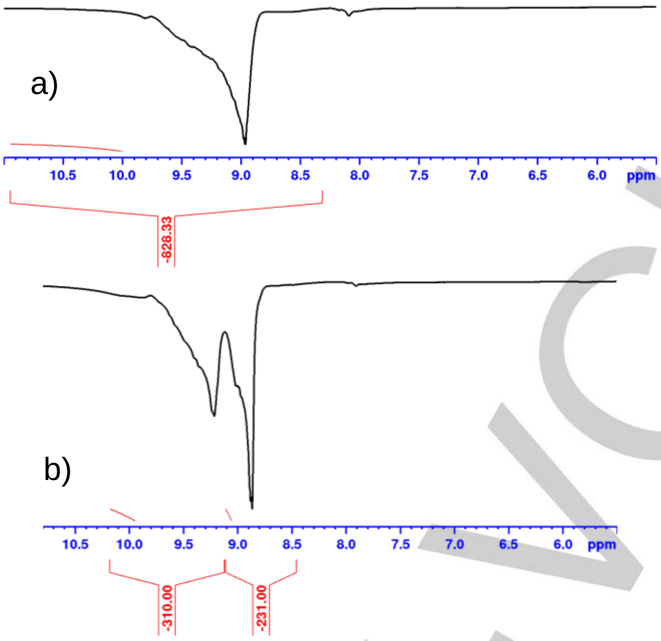

Figure S9. (a) Hyperpolarized pyrazine signal for sample **3a** acquired under SABRE immediately after the polarization transfer step and (b) 30 seconds after the polarization transfer step when complete separation has occurred.

Sample 3b: 1-fold excess pyrazine + Na_2CO_3

A sample was prepared using 1-fold excess of ligand relative to 5 mM of Ir dissolved in a mixture of 0.3 ml $CDCl_3$ and 0.3 D_2O. Na_2CO_3 was added as a separation agent. When examining the result obtained after acquiring a spectrum under Boltzmann equilibrium conditions, it can be seen that, similarly to the data presented above, that chemical shift separation of the pyrazine resonance as a function of solvent is seen.
This chemical shift difference is preserved in the ^1H hyperpolarized spectrum that is acquired after the mixture is allowed to separate. However, as Na_2CO_3 is a much milder base (pK_b 3.67) when compared to NaOH (pK_b 0.2). The two substrate resonances appear much narrower and closer together (Figure S10) with Na_2CO_3.

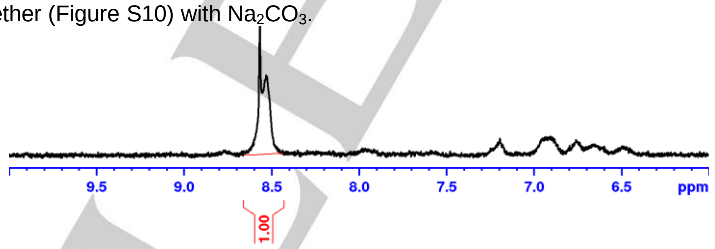

Figure S10. ^1H NMR spectrum of sample **3b** acquired under thermal equilibrium conditions (x8).

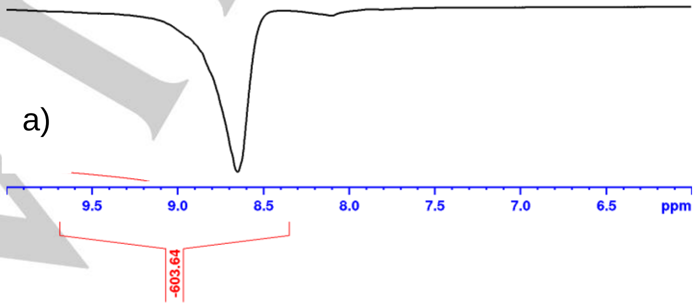

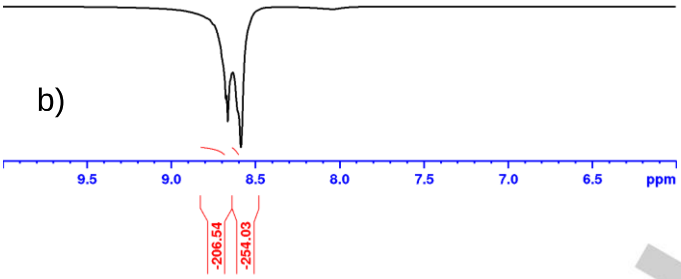

Figure S11. ^{1}H NMR spectrum of sample **3b**, hyperpolarized under SABRE, (a) acquired immediately after the polarization transfer step and (b) 30 seconds after the polarization transfer step, when complete separation has occurred.

Sample 3c: 1-fold excess pyrazine + $NaHCO_3$

A sample was prepared using 1-fold excess of ligand relative to 5 mM of Ir dissolved in a mixture of 0.3 ml $CDCl_3$ and 0.3 D_2O. $NaHCO_3$ was then added as the separation agent. When examining the result obtained after acquiring a spectrum under Boltzmann equilibrium conditions, a chemical shift separation similar to the examples presented above can be detected.

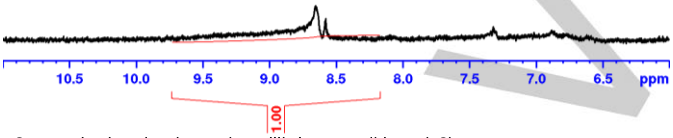

Figure S12. ^{1}H NMR spectrum of sample **3c** acquired under thermal equilibrium conditions (x8).

The analogous spectrum acquired under hyperpolarized conditions, after the mixture was allowed to separate, exhibits several resonances instead of the two observed previously (Figure S13).

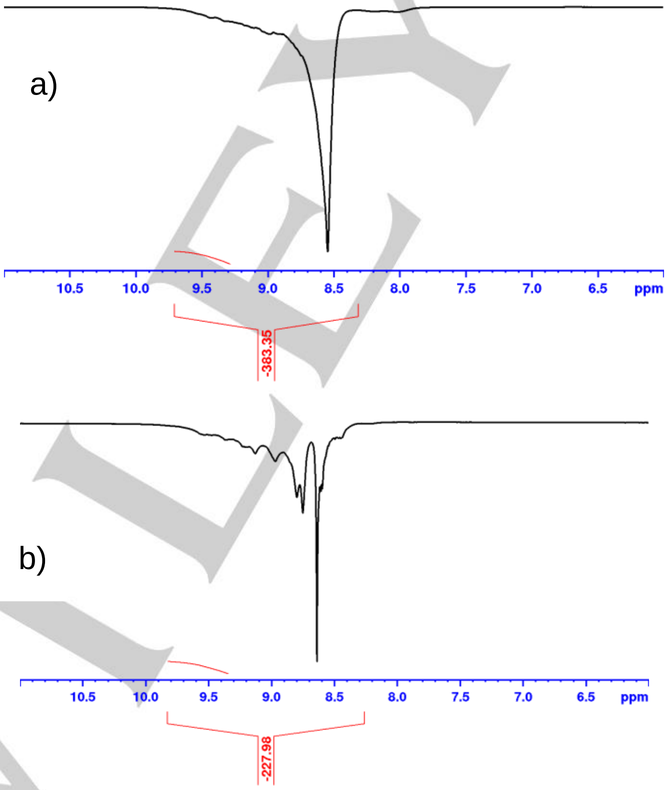

Figure S13. ^{1}H NMR spectrum of sample **3c**, hyperpolarized under SABRE, (a) acquired immediately after the polarization transfer step and (b) 30 seconds after the polarization transfer step which suggests that $NaHCO_3$ takes longer to separate.

Sample 3d: 1-fold excess pyrazine + CH_3COO^- Na^+

A sample was prepared using 1-fold excess of ligand relative to 5 mM of Ir dissolved in a mixture of 0.3 ml $CDCl_3$ and 0.3 D_2O. $NaCO_2CH_3$ was then added as the separation agent. The use of the acidic additive leads to a difference in the chemical shifts of pyrazine in the two solvents.

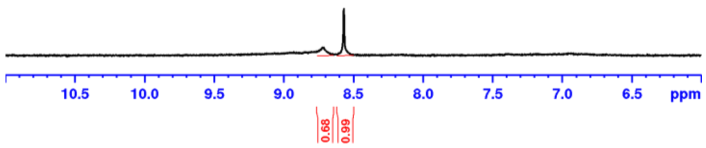

Figure S14. ^{1}H NMR spectrum of sample **3d** acquired under thermal equilibrium conditions (x8).

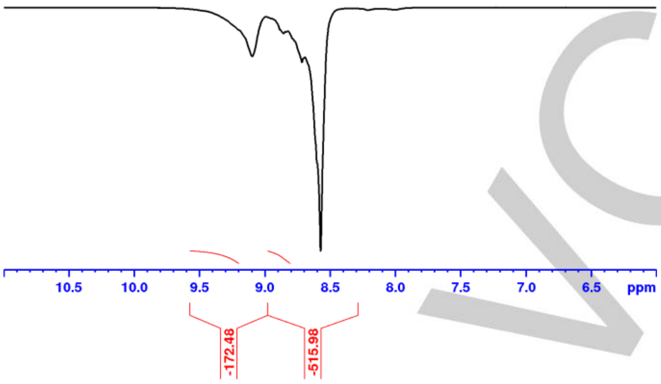

Figure S15. ^{1}H NMR spectrum of sample **3d,** hyperpolarized under SABRE, acquired when complete separation has occurred after 10 seconds.

Sample: 1-fold excess pyrazine + CH_3COO^- NH_4^+ (3e)

A sample was prepared using 1-fold excess of ligand relative to 5 mM of Ir dissolved in a mixture of 0.3 ml $CDCl_3$ and 0.3 D_2O. $NH_4CO_3CH_3$ was added as a separation agent. The ^{1}H spectrum acquired under thermal equilibrium conditions exhibits two sharp resonances accompanied by a very broad peak located downfield (Figure S16).

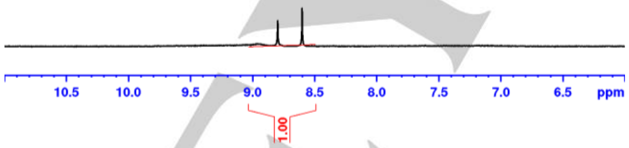

Figure S16. ^{1}H NMR spectrum of sample **3e** acquired under thermal equilibrium conditions.

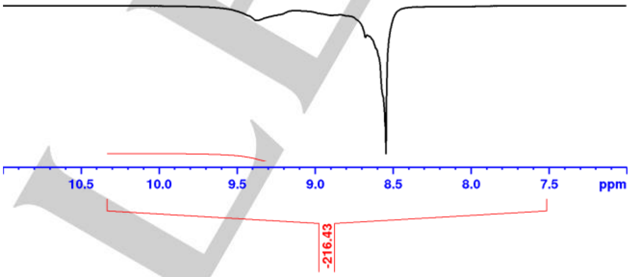

Figure S17. ^{1}H NMR spectrum of **3e** hyperpolarized under SABRE, acquired 30 seconds after the polarization transfer step, when complete separation has occurred.

Sample 3f: 1-fold excess pyrazine + Cl^- NH_4^+

A sample was prepared using 1-fold excess of ligand relative to 5 mM of Ir dissolved in a mixture of 0.3 ml $CDCl_3$ and 0.3 D_2O. NH_4Cl was added as the phase separation agent. The ^{1}H spectrum acquired under thermal equilibrium conditions exhibits two resonances (Figure S18) for the two phases. Phase separation after shaking proceeds as detailed in Figures S18b and S18c.

WILEY-VCH

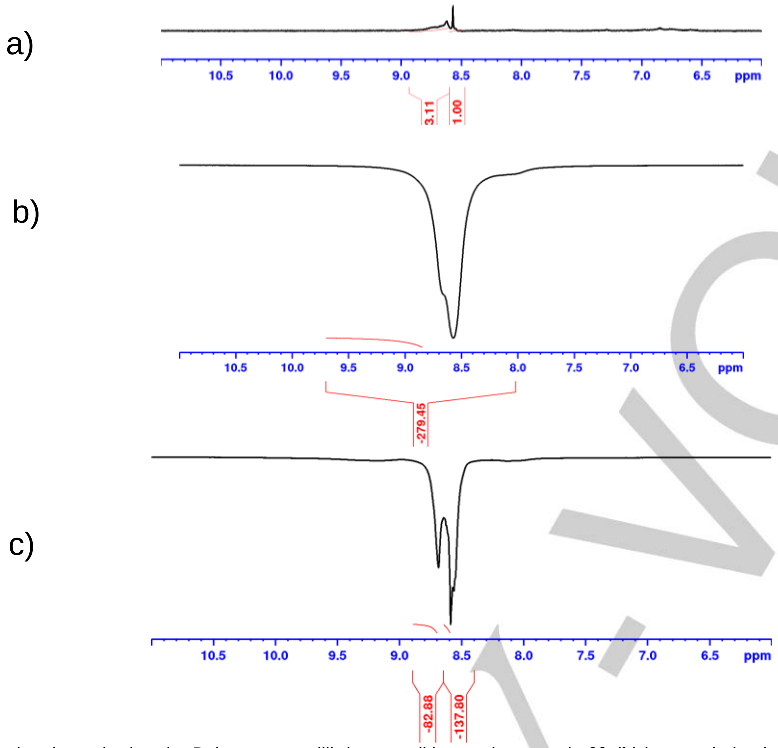

Figure S18. (**a**) Pyrazine signal acquired under Boltzmann equilibrium conditions using sample **3f**, (**b**) hyperpolarized signal under SABRE, acquired immediately after the polarization transfer step and (**c**) signal recorded 30 seconds after the polarization transfer step when complete separation has occurred.

Sample 3g: 17-fold excess pyrazine + NH_4Cl + NaCl

A sample was prepared using 17-fold excess of ligand relative to 5 mM of Ir dissolved in a mixture of 0.3 ml $CDCl_3$ and 0.3 D_2O. NH_4Cl and NaCl were added to promote phase separation. The ^1H spectrum acquired under thermal equilibrium conditions exhibits two sharp resonances accompanied by a very broad peak located downfield (Figure S19). While a poor polarization level is observed, the phase separation time is also slow.

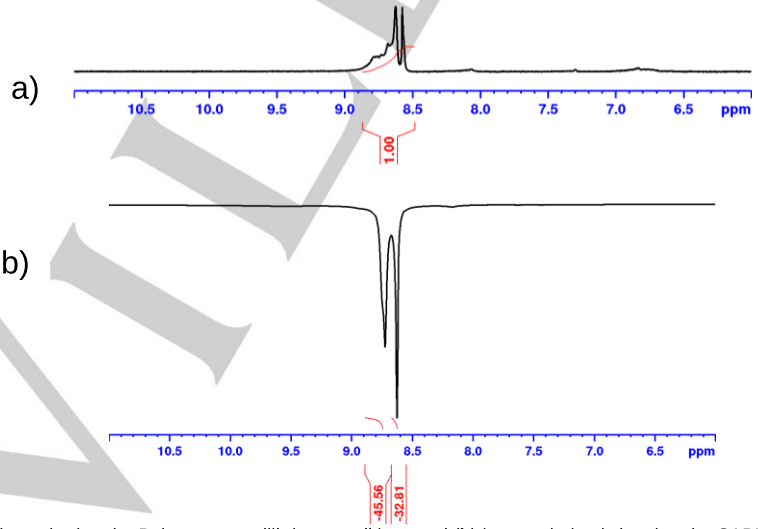

Figure S19. (**a**) Pyrazine signal acquired under Boltzmann equilibrium conditions and (**b**) hyperpolarized signal under SABRE, acquired 30 seconds after the polarization transfer step when complete separation has occurred.

WILEY-VCH

2. 1D MRI results

2.1 SABRE hyperpolarization of pyrazine in $CDCl_3/D_2O$ mixtures

Figures S20 and S21 reflect sample **1g** (without salt) and present a series of time-resolved 1D projections which detail how phase separation proceeds. Figure S20 details a projection of the ^2H distribution (left) and reflects the dominant D_2O solvent. Figure S21 (left) shows the weak ^1H response of pyrazine, acquired under thermal polarization conditions. Figure S21 (right) shows the results of shaking this sample under *p*-H_2. Hyperpolarization in the aqueous phase results even though the $CDCl_3$ doping is minimal.

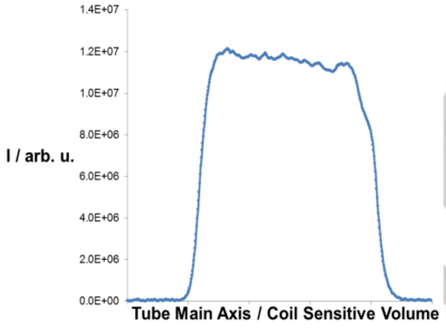

Figure S20. 1D projection of the ^2H NMR signal showing the distribution of solvents in the NMR tube in sample **1g** prior to the shaking process.

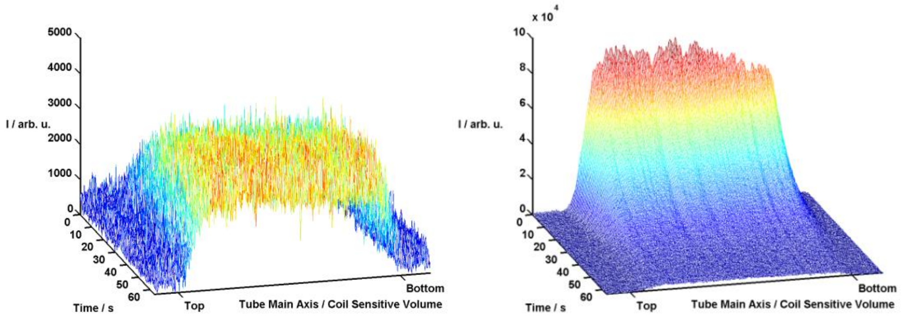

Figure S21. 1D projection of the pyrazine signal after shaking the sample for 10 seconds as a function of time. Left: thermal equilibrium conditions. Right: SABRE hyperpolarized.

2.2 SABRE hyperpolarization of pyrazine in $CDCl_3/D_2O$ mixtures in the presence of NaCl

Figures S22-S37 reflect samples **2b**-**2i** and present a series of time-resolved 1D projections which detail how phase separation is affected by added NaCl and the ratio of $CDCl_3$ to D_2O. These traces reveal that the best phase separation time resulted when the $CDCl_3$:D_2O ratio was 1:1 and 1 mg of NaCl was present. A common format is employed for each sample wherein the first figure shows a projection of the ^2H distribution (left) and reflects the two solvents. The right trace shows the weak ^1H response of pyrazine, acquired under thermal polarization conditions. The second figure for each sample shows the results of shaking this sample under normal-H_2 (a 1:3 mixture of *para* and *ortho*), left trace and the corresponding SABRE response (right trace).

Sample 2b: 1-fold excess pyrazine dissolved in 0.4 ml $CDCl_3$ + 0.2 ml D_2O + 1 mg NaCl

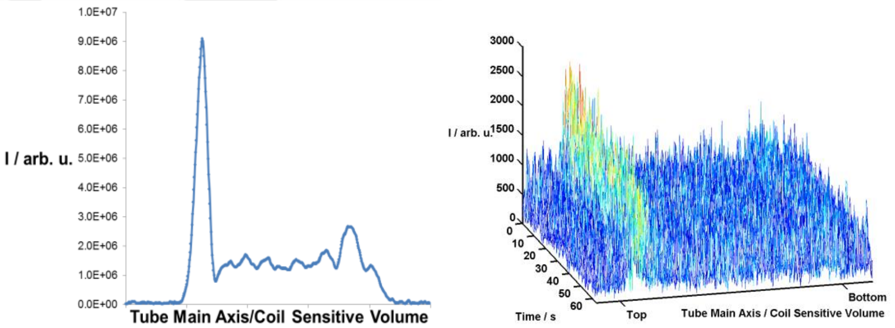

Figure S22. Left: 1D projection of ^2H NMR signal showing the distribution of solvents in the NMR tube prior to the shaking process. Right: 1D projections of the ^1H NMR signal as a function of time.

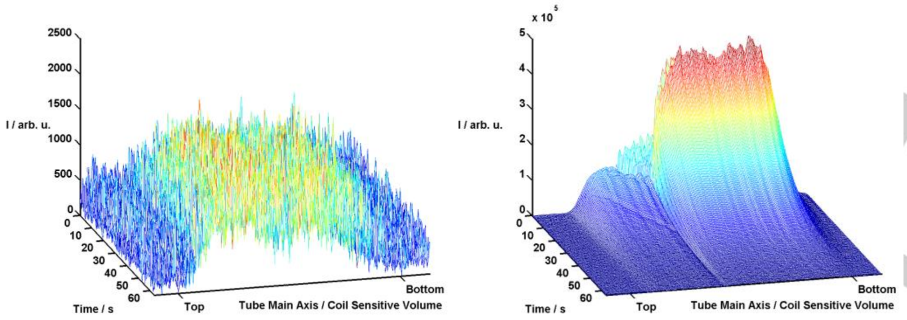

Figure S23. 1D projection of the pyrazine signal after shaking the sample for 10 seconds as a function of time. Left: thermal equilibrium conditions. Right: SABRE hyperpolarized.

Sample 2c: 1-fold excess pyrazine dissolved in 0.3 ml $CDCl_3$ + 0.3 ml D_2O + 1 mg NaCl

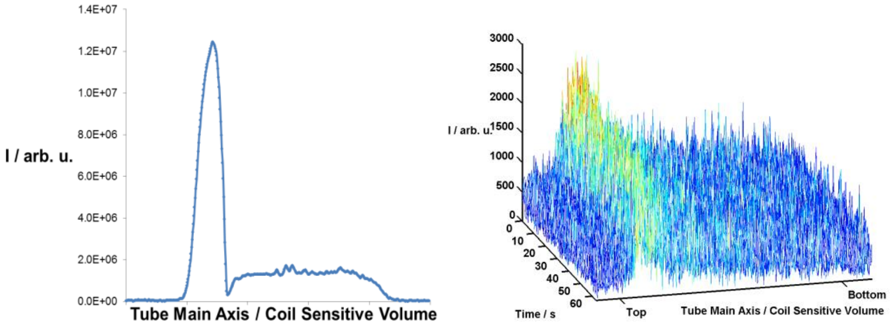

Figure S24. Left: 1D projection of the ^{2}H signal showing the distribution of solvents in the NMR tube prior to the shaking process. Right: 1D projections of the ^{1}H NMR signal as a function of time.

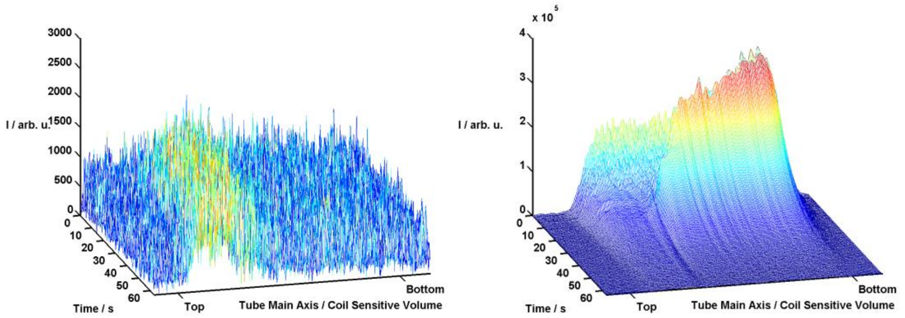

Figure S25. 1D projection of the pyrazine signal after shaking the sample for 10 seconds as a function of time. Left: thermal equilibrium conditions. Right: SABRE hyperpolarized.

Sample 2d: 1-fold excess pyrazine dissolved in 0.2 ml $CDCl_3$ + 0.4 ml D_2O + 1 mg NaCl

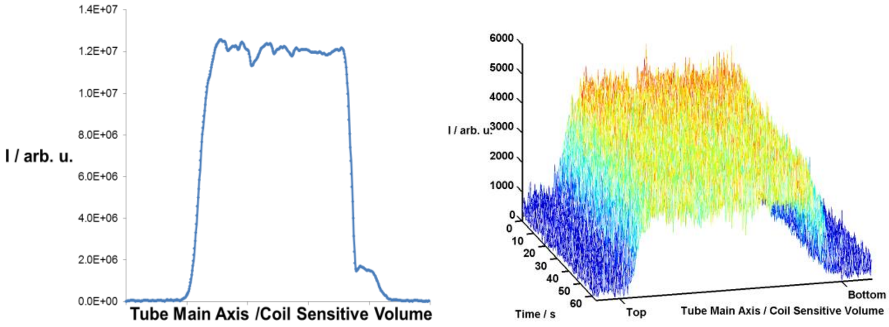

Figure S26. Left: 1D projection of ^{2}H NMR signal showing the distribution of solvents in the NMR tube prior to the shaking process. Right: 1D projections of the ^{1}H NMR signal as a function of time.

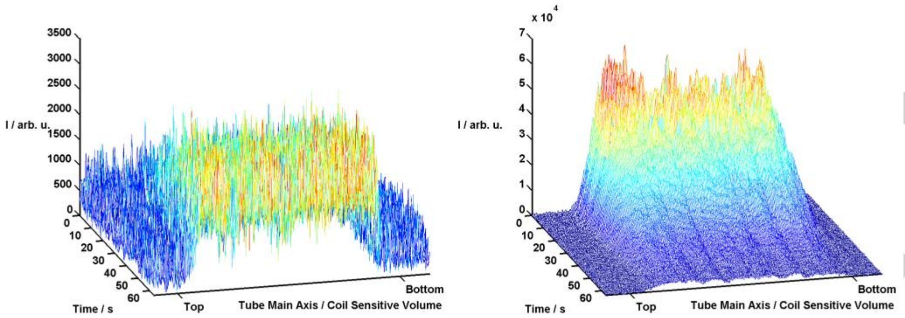

Figure S27. 1D projection of the pyrazine signal after shaking the sample for 10 seconds as a function of time. Left: thermal equilibrium conditions. Right: SABRE hyperpolarized.

Sample 2e: 1-fold excess pyrazine dissolved in 0.1 ml $CDCl_3$ + 0.5 ml D_2O + 1 mg NaCl

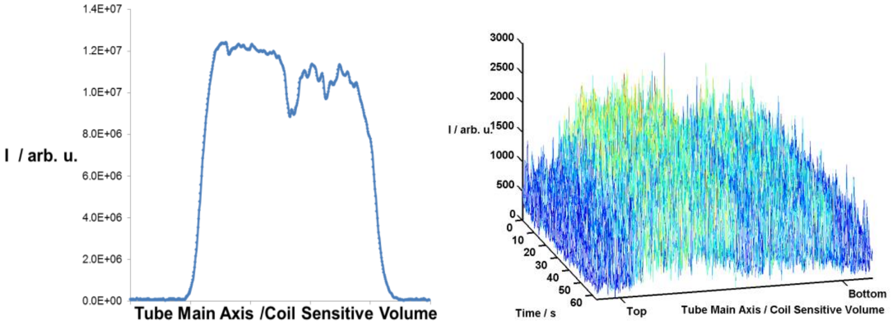

Figure S28. Left: 1D projection of ^{2}H signal showing the distribution of solvents in the NMR tube prior to the shaking process. Right: 1D projections of the ^{1}H signal as a function of time.

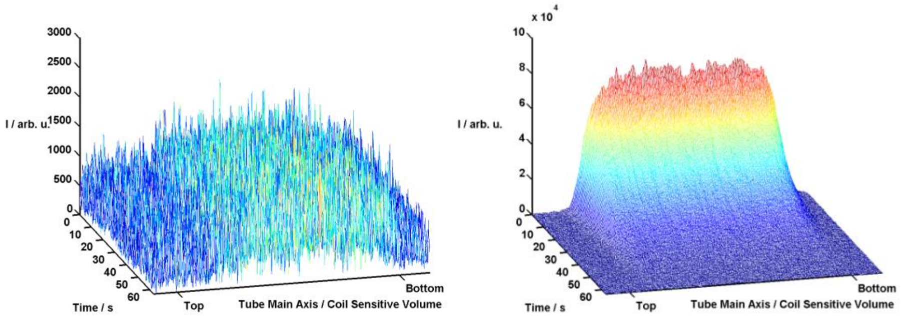

Figure S29. 1D projection of the pyrazine signal after shaking the sample for 10 seconds as a function of time. Left: thermal equilibrium conditions. Right: SABRE hyperpolarized.

Sample 2f: 1-fold excess pyrazine dissolved in 0.25 ml $CDCl_3$ + 0.35 ml D_2O + 0.5 mg NaCl

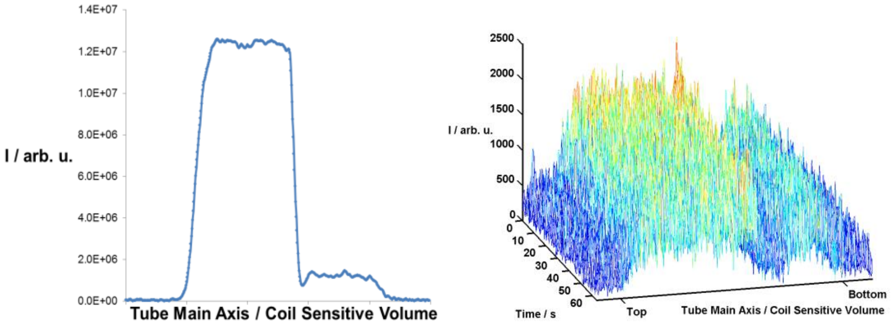

Figure S30. Left: 1D projection of ^{2}H signal showing the distribution of solvents in the NMR tube prior to the shaking process. Right: 1D projections of the ^{1}H signal as a function of time.

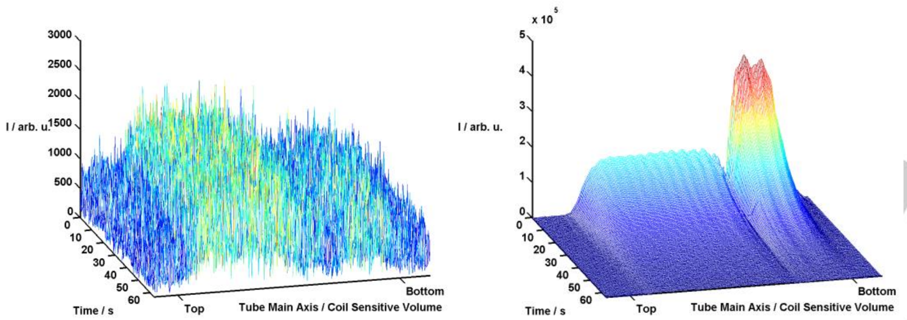

Figure S31. 1D projection of the pyrazine signal after shaking the sample for 10 seconds as a function of time. Left: thermal equilibrium conditions. Right: SABRE hyperpolarized.

Sample 2g: 17-fold excess pyrazine dissolved in 0.3 ml $CDCl_3$ + 0.3 ml D_2O + 1 mg NaCl

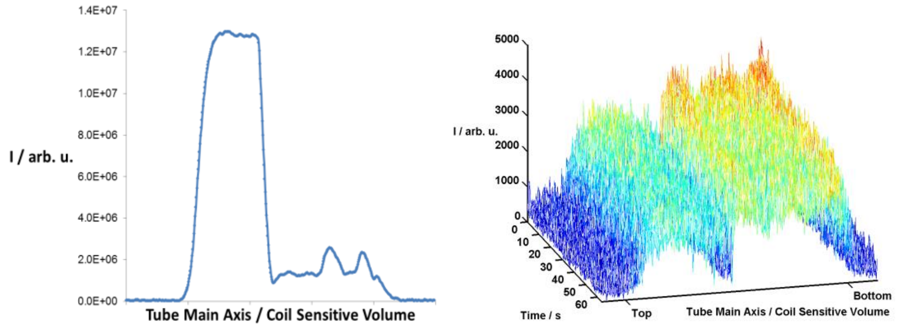

Figure S32. Left: 1D projection of ^{2}H signal showing the distribution of solvents in the NMR tube prior to the shaking process. Right: 1D projections of the ^{1}H signal as a function of time.

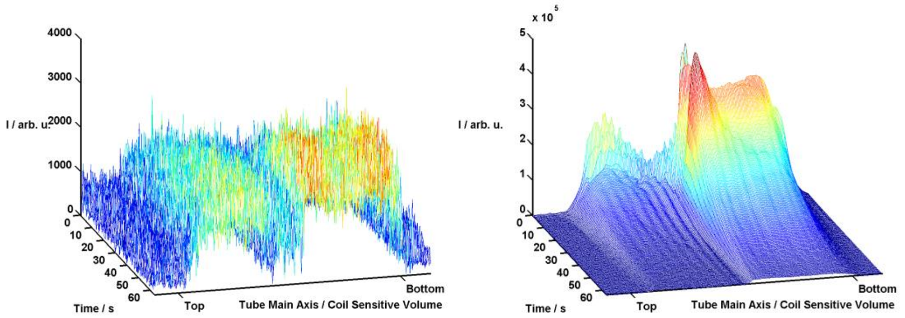

Figure S33. 1D projection of the pyrazine signal after shaking the sample for 10 seconds as a function of time. Left: thermal equilibrium conditions. Right: SABRE hyperpolarized.

Sample 2h: 17-fold excess pyrazine dissolved in 0.3 ml $CDCl_3$ + 0.3 ml D_2O + 2 mg NaCl

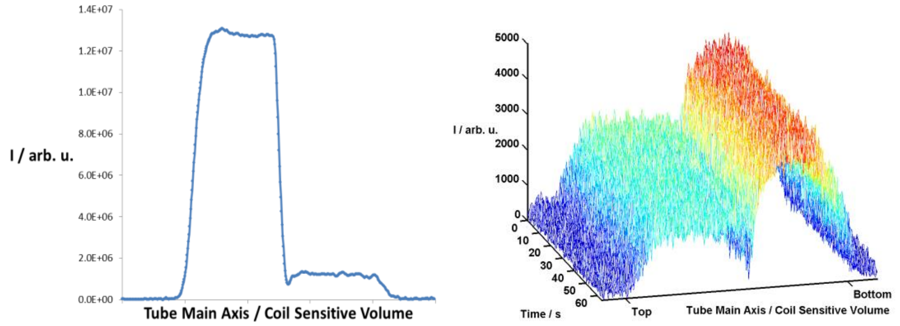

Figure S34. Left: 1D projection of ^{2}H signal showing the distribution of solvents in the NMR tube prior to the shaking process. Right: 1D projections of the ^{1}H signal as a function of time.

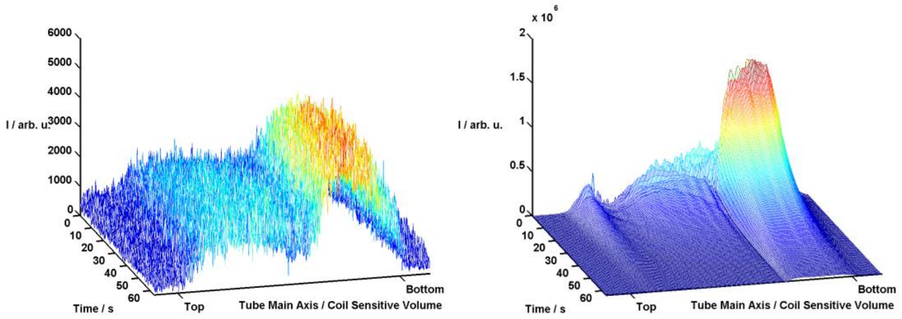

Figure S35. 1D projection of the pyrazine signal after shaking the sample for 10 seconds as a function of time. Left: thermal equilibrium conditions. Right: SABRE hyperpolarized.

Sample 2i: 17-fold excess pyrazine dissolved in 0.3 ml $CDCl_3$ + 0.3 ml D_2O + 2mg NaCl + 50 µl EtOD

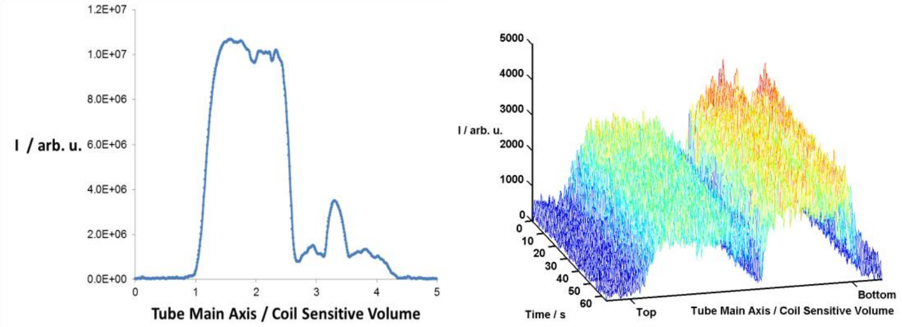

Figure S36. Left: 1D projection of ^{2}H signal showing the distribution of solvents in the NMR tube prior to the shaking process. Right: 1D projections of the ^{1}H signal as a function of time.

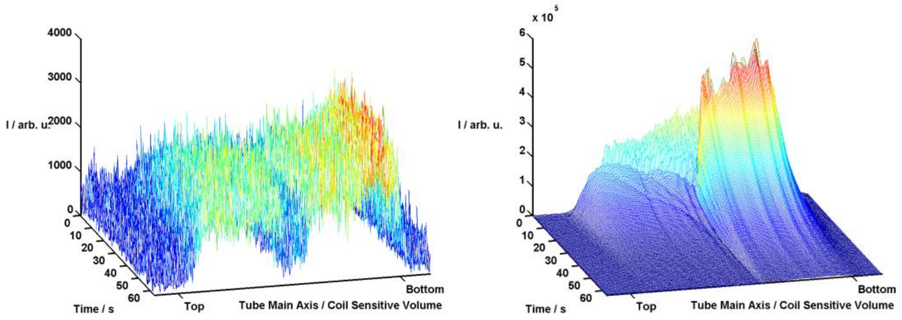

Figure S37. 1D projection of the pyrazine signal after shaking the sample for 10 seconds as a function of time. Left: thermal equilibrium conditions. Right: SABRE hyperpolarized.

2.3 SABRE hyperpolarization of pyrazine in $CDCl_3$/D_2O mixtures in the presence of other salts

Figures S38-S52 reflect samples **3a**-**3h** and present a series of time-resolved 1D projections which detail the impact of the salt on the phase separation time. The ratio of $CDCl_3$ to D_2O is maintained at 1:1. These traces reveal that the best agent is NaCl. A common format is employed for each sample wherein the first figure shows a projection of the ^{2}H distribution (left) and reflects the two solvents. The right trace shows the weak ^{1}H response of pyrazine, acquired under thermal polarization conditions. The second figure for each sample shows the results of shaking this sample under normal-H_2 (a 1:3 mixture of *para* and *ortho*), left trace and the corresponding SABRE response (right trace).

Sample 3a: 1-fold excess pyrazine dissolved in 0.3 ml $CDCl_3$ + 0.3 ml D_2O + 0.35 mg NaOH

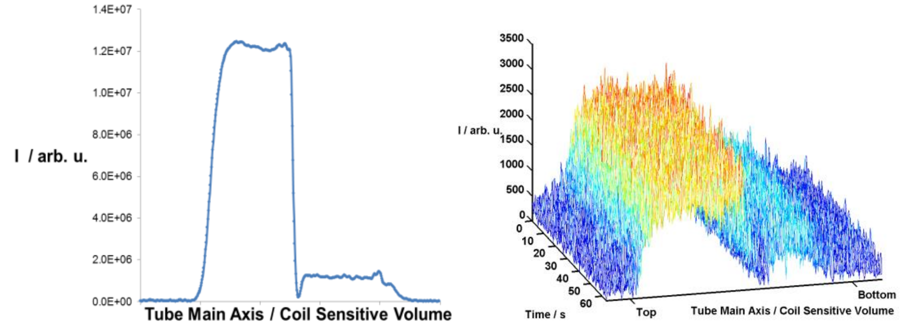

Figure S38. Left: 1D projection of ^{2}H signal showing the distribution of solvents in the NMR tube prior to the shaking process. Right: 1D projections of the ^{1}H signal as a function of time.

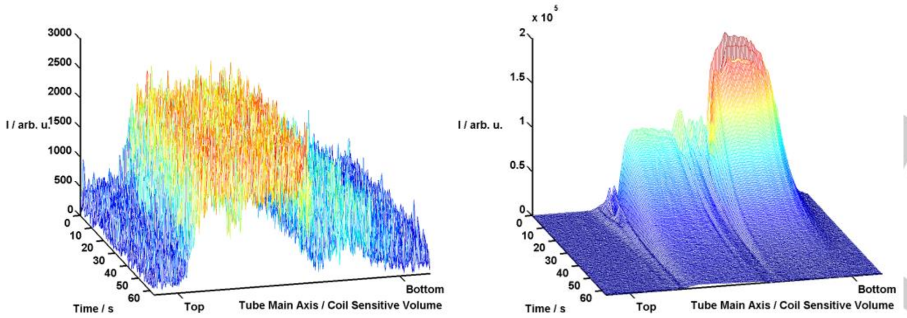

Figure S39. 1D projection of the pyrazine signal after shaking the sample for 10 seconds as a function of time. Left: thermal equilibrium conditions. Right: SABRE hyperpolarized.

Sample 3b: 1-fold excess pyrazine dissolved in 0.3 ml $CDCl_3$ + 0.3 ml D_2O + 1mg Na_2CO_3

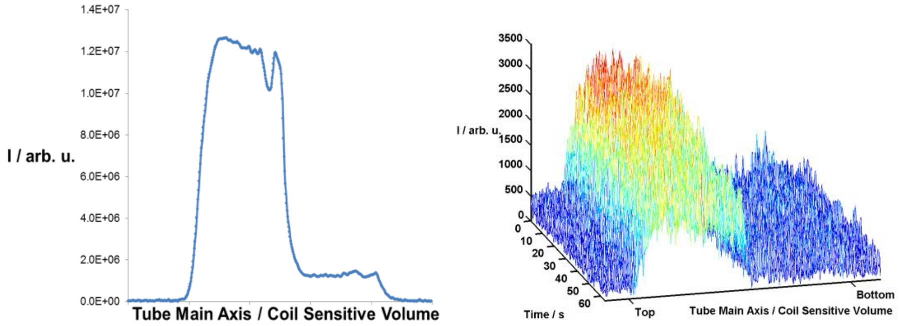

Figure S40. Left: 1D projection of ^{2}H signal showing the distribution of solvents in the NMR tube prior to the shaking process. Right: 1D projections of the ^{1}H signal as a function of time.

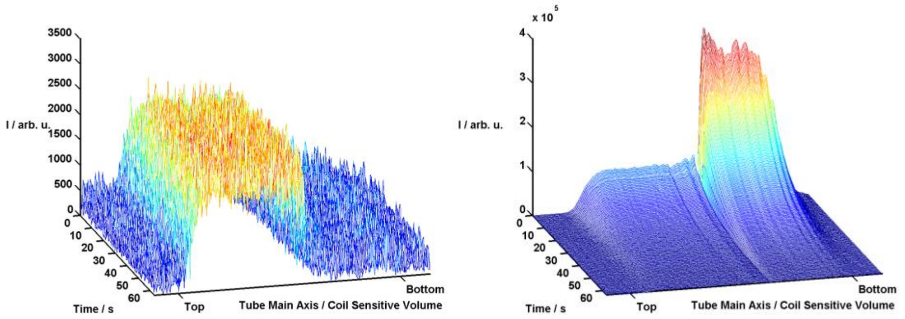

Figure S41. 1D projection of the pyrazine signal after shaking the sample for 10 seconds as a function of time. Left: thermal equilibrium conditions. Right: SABRE hyperpolarized.

Sample 3c: 1-fold excess pyrazine dissolved in 0.3 ml $CDCl_3$ + 0.3 ml D_2O + 0.7mg $NaHCO_3$

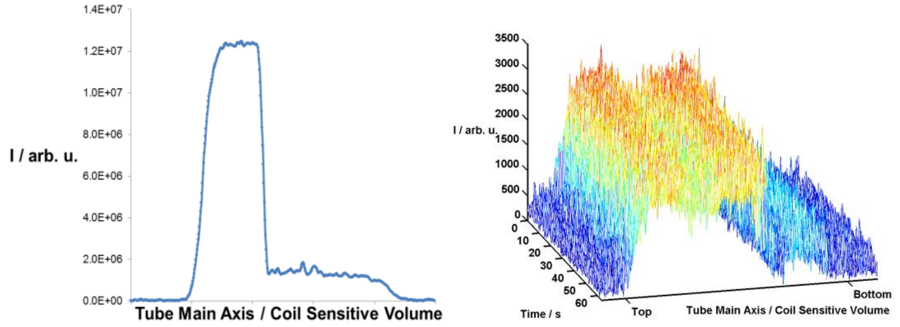

Figure S42. Left: 1D projection of ^{2}H signal showing the distribution of solvents in the NMR tube prior to the shaking process. Right: 1D projections of the ^{1}H signal as a function of time.

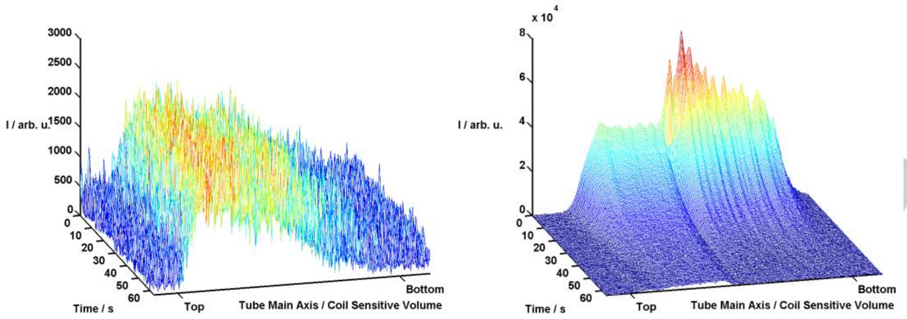

Figure S43. 1D projection of the pyrazine signal after shaking the sample for 10 seconds as a function of time. Left: thermal equilibrium conditions. Right: SABRE hyperpolarized.

Sample 3d: 1-fold excess pyrazine dissolved in 0.3 ml $CDCl_3$ + 0.3 ml D_2O + 0.57 mg $Na^+CH_3COO^-$

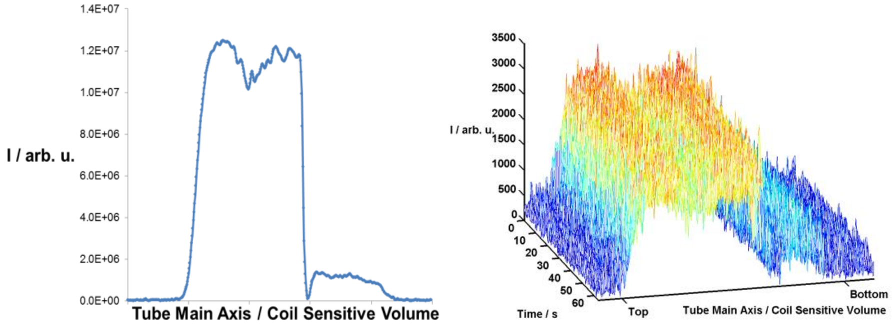

Figure S44. Left: 1D projection of ^{2}H signal showing the distribution of solvents in the NMR tube prior to the shaking process. Right: 1D projections of the ^{1}H signal as a function of time.

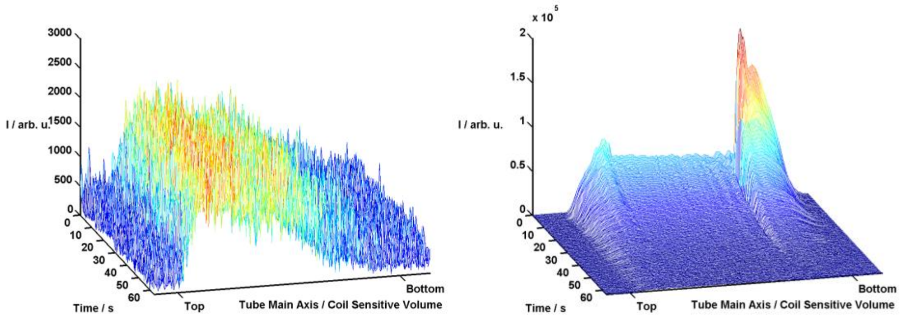

Figure S45. 1D projection of the pyrazine signal after shaking the sample for 10 seconds as a function of time. Left: thermal equilibrium conditions. Right: SABRE hyperpolarized.

Sample 3e: 1-fold excess pyrazine dissolved in 0.3 ml $CDCl_3$ + 0.3 ml D_2O + 0.6 mg $NH_4^+CH_3COO^-$

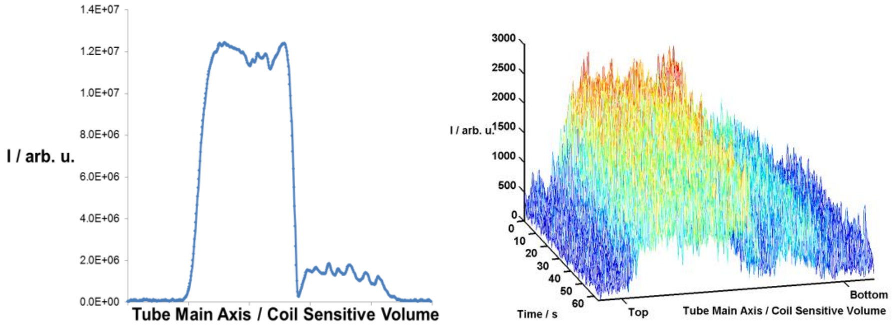

Figure S46. Left: 1D projection of ^{2}H signal showing the distribution of solvents in the NMR tube prior to the shaking process. Right: 1D projections of the ^{1}H signal as a function of time.

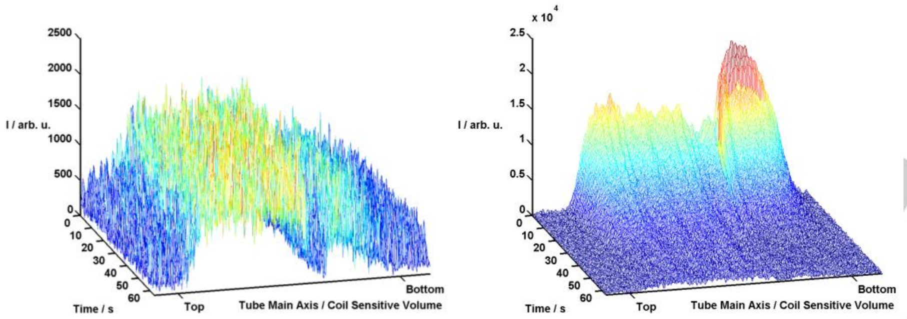

Figure S47. 1D projection of the pyrazine signal after shaking the sample for 10 seconds as a function of time. Left: thermal equilibrium conditions. Right: SABRE hyperpolarized.

Sample 3f: 1-fold excess pyrazine dissolved in 0.3 ml $CDCl_3$ + 0.3 ml D_2O + 0.5 mg NH_4Cl

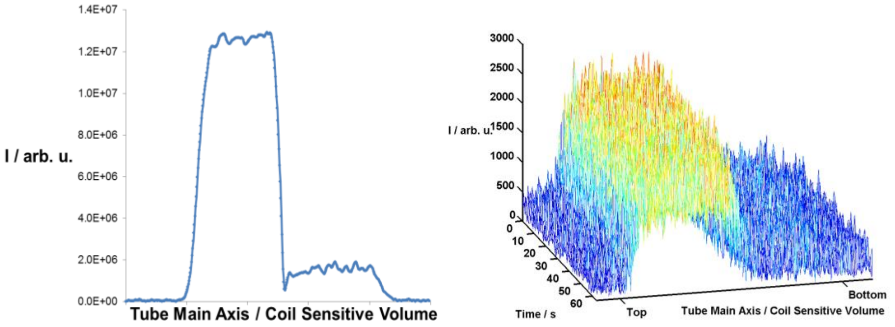

Figure S48. Left: 1D projection of ^2H signal showing the distribution of solvents in the NMR tube prior to the shaking process. Right: 1D projections of the ^1H signal as a function of time.

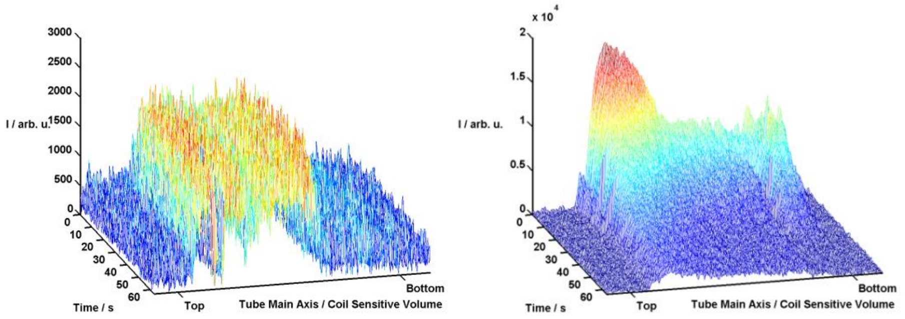

Figure S49. 1D projection of the pyrazine signal after shaking the sample for 10 seconds as a function of time. Left: thermal equilibrium conditions. Right: SABRE hyperpolarized.

Sample 3g: 17-fold excess pyrazine dissolved in 0.3 ml $CDCl_3$ + 0.3 ml D_2O + 0.5 mg NH_4Cl + 1 mg NaCl

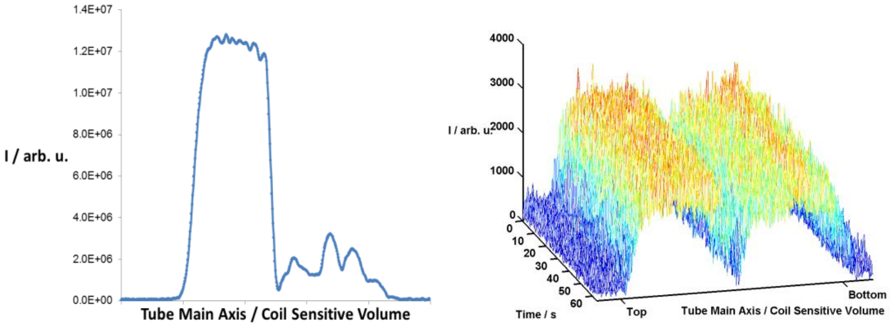

Figure S50. Left: 1D projection of ^2H signal showing the distribution of solvents in the NMR tube prior to the shaking process. Right: 1D projections of the ^1H signal as a function of time.

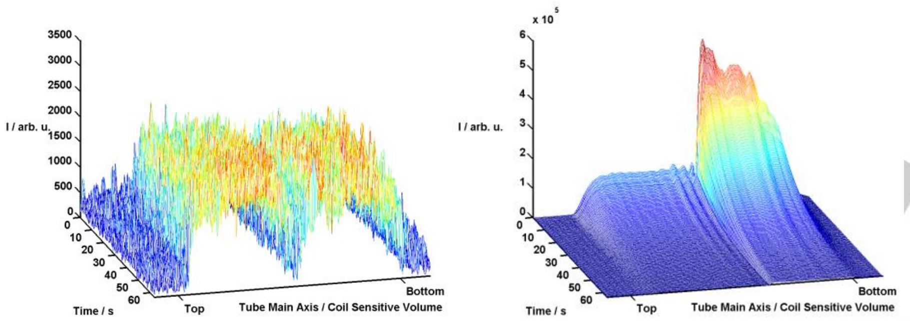

Figure S51. 1D projection of the pyrazine signal after shaking the sample for 10 seconds as a function of time. Left: thermal equilibrium conditions. Right: SABRE hyperpolarized.

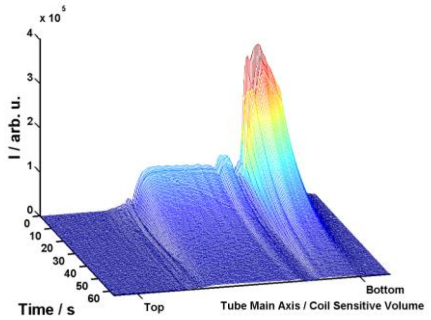

Figure S52. 1D projection of the pyrazine signal after shaking the sample for 10 seconds as a function of time measured after 20 s from the shaking process and after adding another 4.5 mg NH_4Cl.

2.4 SABRE hyperpolarization of other substrates in $CDCl_3/D_2O$ mixtures

In order to demonstrate that the approach of CASH SABRE is widely extendable to a whole range of substrates, we have performed hyperpolarization experiments on a set of samples prepared using a 1:1 organic: aqueous ratio (0.3 ml : 0.3 ml) and a series of ligands which present significant interest in terms of biomedical applications. A 5 mM catalyst loading was employed in conjunction with 20 mM of the agent and 0.16w/v NaCl. The agents analyzed are presented in Scheme S1. Figures S53-S56 present ^{1}H NMR spectra that detail the SABRE hyperpolarization of the ^{1}H response of these samples and confirm the wider applicability of CASH SABRE. The very high polarization level resulting for d_2-methylnicotinate is particularly notable. Figures S57-S68 detail a series of time-resolved 1D projections on these samples to illustrate how phase separation proceeds in a similar way to that presented earlier.

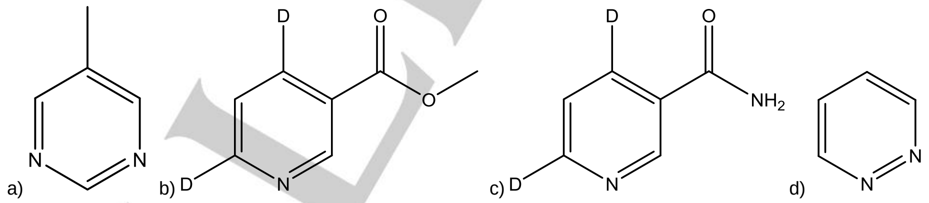

Scheme S1. Substrates tested to prove the wider applicability of the CASH SABRE method: a) 5-methylpyrimidine, b) 4,6-d_2-methyl nicotinate, c) 4,6-d_2-nicotinamide and d) pyridazine.

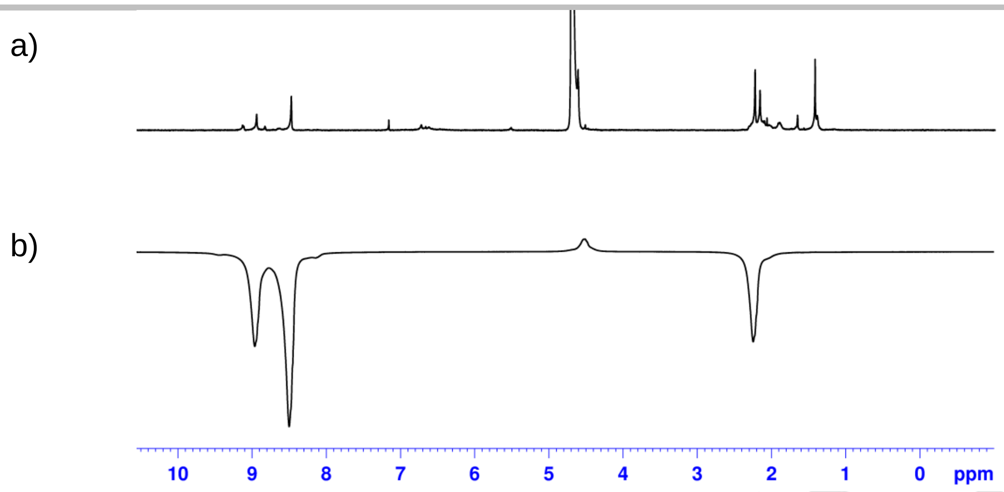

Figure S53. ^{1}H NMR spectra of a sample of 5-methylpyrimidine collected a) under thermal conditions (vertical expansion x16 relative to (b) and b) 10 seconds after CASH-SABRE hyperpolarization (hyperpolarization level leads to 340-fold enhancement of the δ 8.96 resonance).

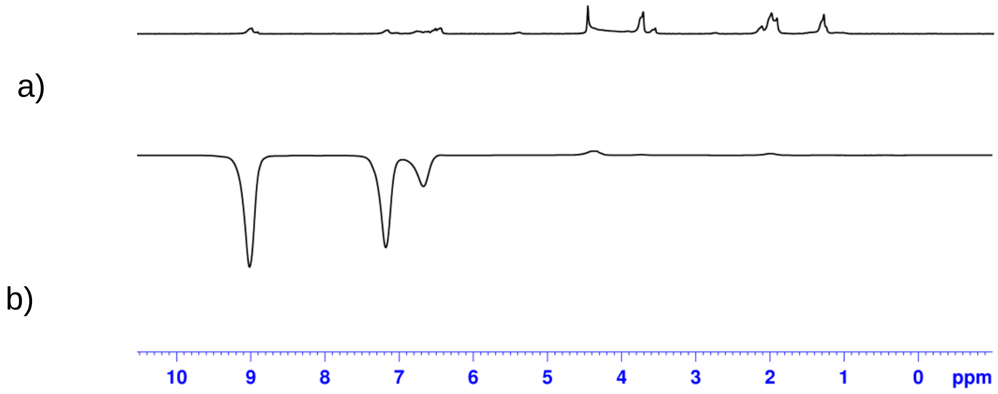

Figure S54. ^{1}H NMR spectra of a sample of 4,6-d_2-methyl nicotinate collected a) under thermal conditions (vertical expansion x128 relative to (b) and b) 10 seconds after CASH-SABRE hyperpolarization (hyperpolarization level leads to 3000-fold enhancement of the δ 7.2 resonance).

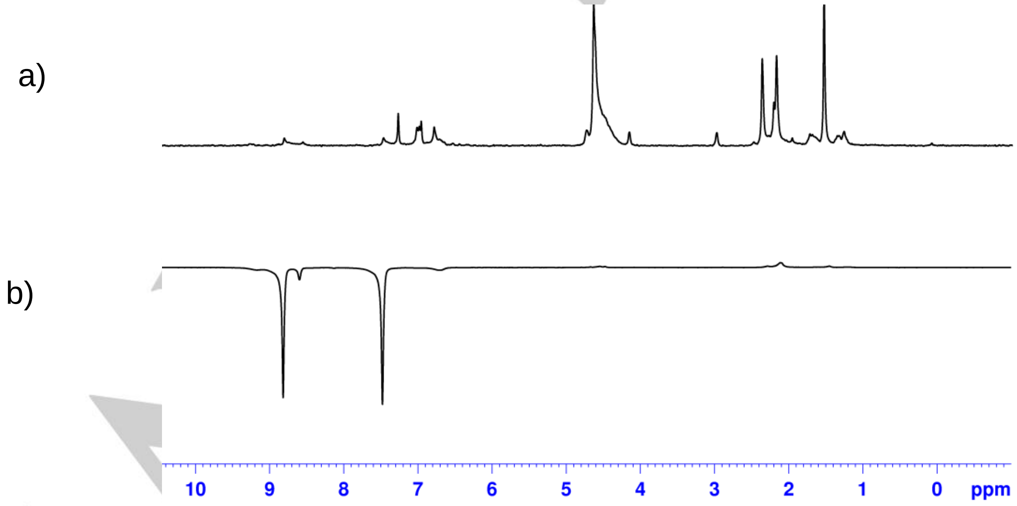

Figure S55. ^{1}H NMR spectra of a sample of 4,6-d_2-nicotinamide collected a) under thermal conditions (vertical expansion x16 relative to (b) and b) 10 seconds after CASH-SABRE hyperpolarization (hyperpolarization level leads to ~260-fold enhancement of the δ 8.8 resonance).

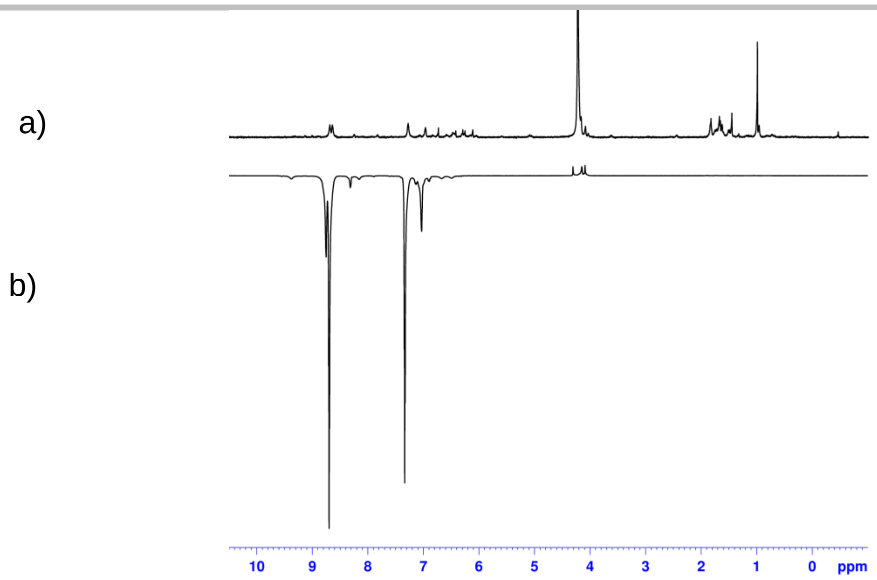

Figure S56. ^{1}H NMR spectra of a sample of pyridazine collected a) under thermal conditions (vertical expansion x32 relative to (b) and b) 10 seconds after CASH-SABRE hyperpolarization (hyperpolarization level leads to a 380-fold enhancement of the δ 8.7 resonance after 10 seconds).

Sample: 1-fold excess 5-methylpyrimidine dissolved in 0.3 ml $CDCl_3$ + 0.3 ml D_2O + 0.5 mg NaCl

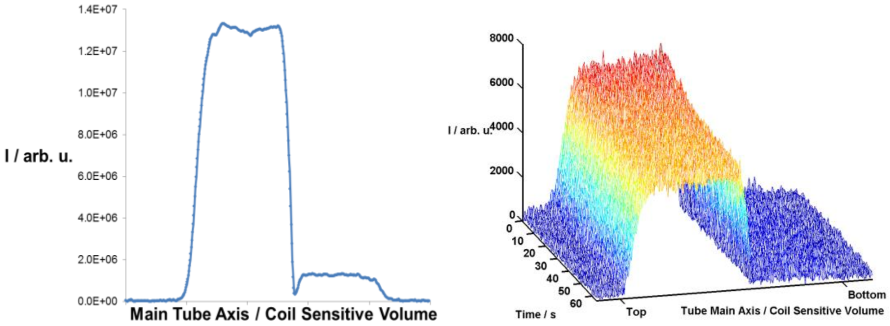

Figure S57. Left: 1D projection of ^{2}H signal showing the distribution of solvents in the NMR tube prior to the shaking process. Right: 1D projections of the ^{1}H signal as a function of time.

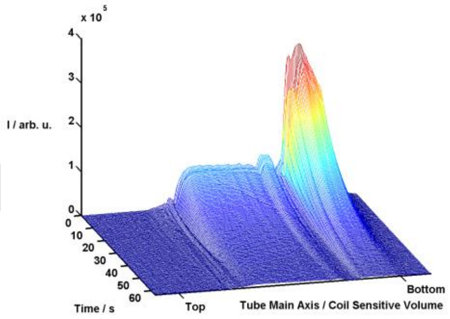

Figure S58. 1D projection of the substrate signal after shaking the sample for 10 seconds as a function of time after adding *p*-H_2.

Sample: 1-fold excess 5-methylpyrimidine dissolved in 0.3 ml $CDCl_3$ + 0.3 ml D_2O + 2 mg NaCl + 20 µl EtOD

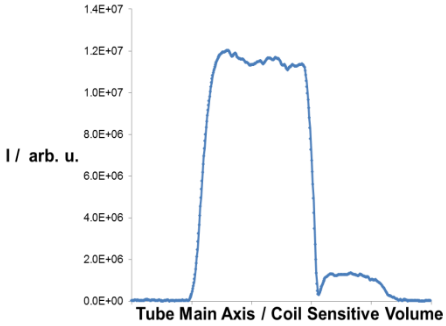

Figure S59. 1D projection of ^{2}H signal showing the distribution of solvents in the NMR tube prior to the shaking process.

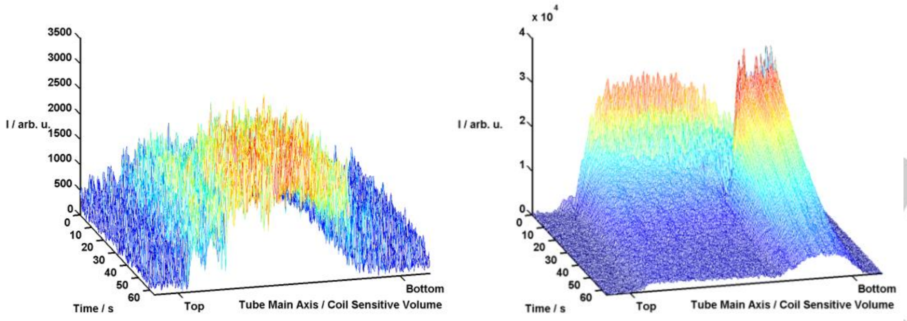

Figure S60. 1D projection of the substrate signal after shaking the sample for 10 seconds as a function of time. Left: thermal equilibrium conditions. Right: SABRE hyperpolarized.

Sample: 1-fold excess 5-methylpyrimidine dissolved in 0.3 ml $CDCl_3$ + 0.3 ml D_2O + 2 mg NaCl + 40 µl EtOD

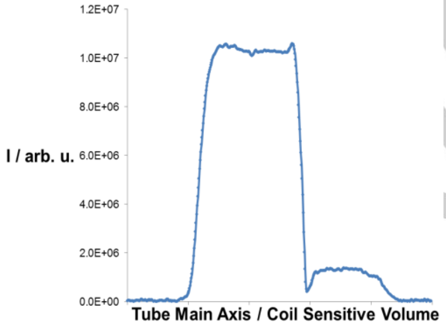

Figure S61. 1D projection of ^{2}H signal showing the distribution of solvents in the NMR tube prior to the shaking process.

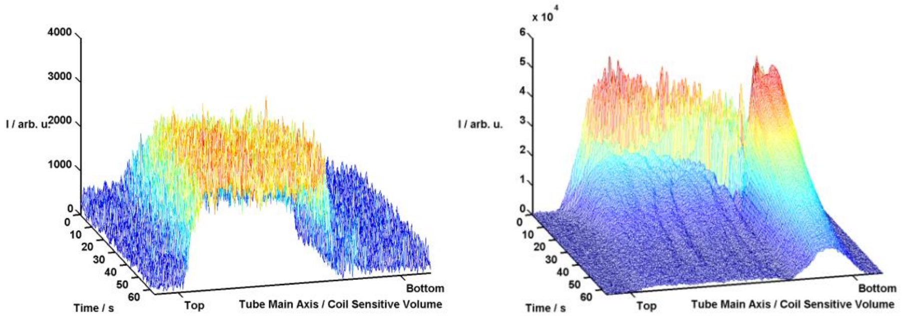

Figure S62. 1D projection of the substrate signal after shaking the sample for 10 seconds as a function of time. Left: thermal equilibrium conditions. Right: SABRE hyperpolarized.

Sample: 1-fold excess pyridazine dissolved in 0.3 ml $CDCl_3$ + 0.3 ml D_2O + 1.0 mg NaCl

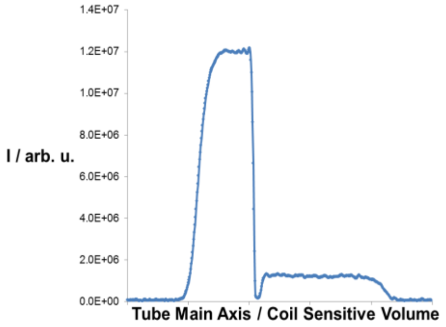

Figure S63. Left: 1D projection of ^{2}H signal showing the distribution of solvents in the NMR tube prior to the shaking process.

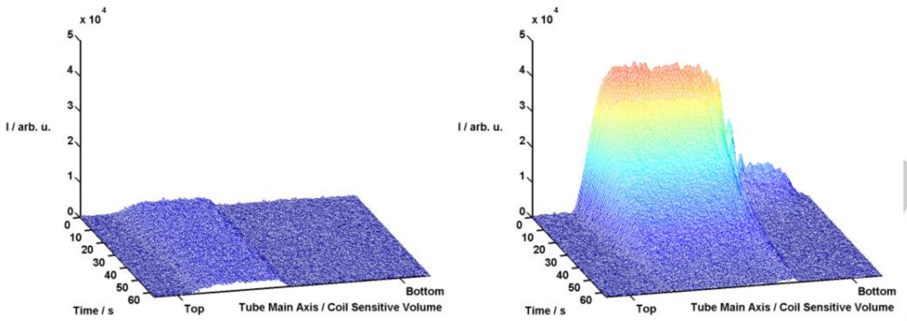

Figure S64. 1D projection of the substrate signal after shaking the sample for 10 seconds as a function of time. Left: thermal equilibrium conditions. Right: SABRE hyperpolarized.

Sample: 1-fold excess *d*$_2$-methylnicotinate dissolved in 0.3 ml $CDCl_3$ + 0.3 ml D_2O + 1.0 mg NaCl

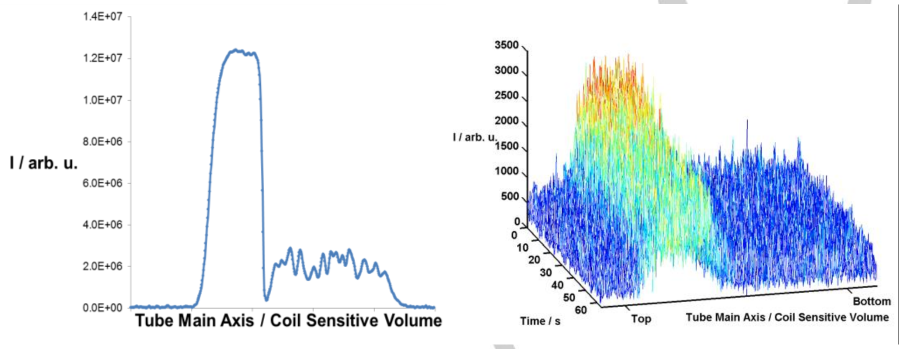

Figure S65. Left: 1D projection of ^{2}H signal showing the distribution of solvents in the NMR tube prior to the shaking process. Right: 1D projections of the ^{1}H signal as a function of time.

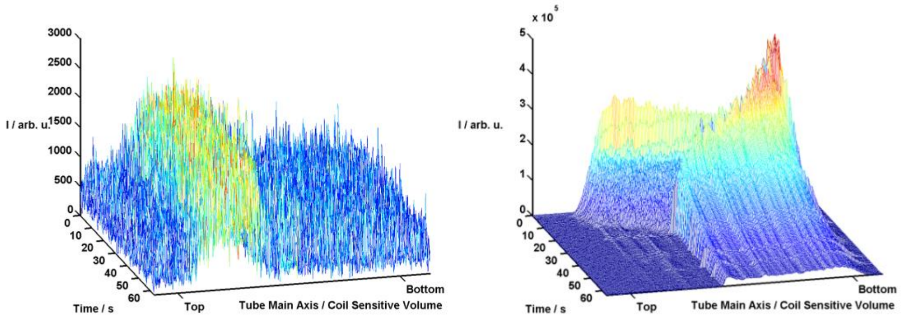

Figure S66. 1D projection of the substrate signal after shaking the sample for 10 seconds as a function of time. Left: thermal equilibrium conditions. Right: SABRE hyperpolarized.

Sample: 1-fold excess 4,6-*d*$_2$-nicotinamide dissolved in 0.3 ml $CDCl_3$ + 0.3 ml D_2O + 1.0 mg NaCl

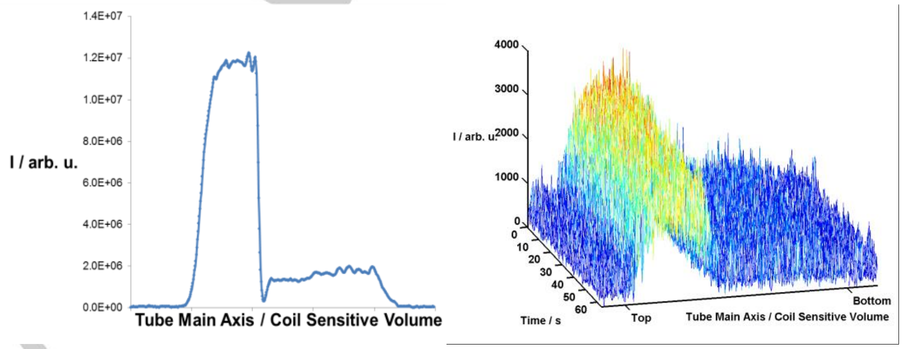

Figure S67. Left: 1D projection of ^{2}H signal showing the distribution of solvents in the NMR tube prior to the shaking process. Right: 1D projections of the ^{1}H signal as a function of time.

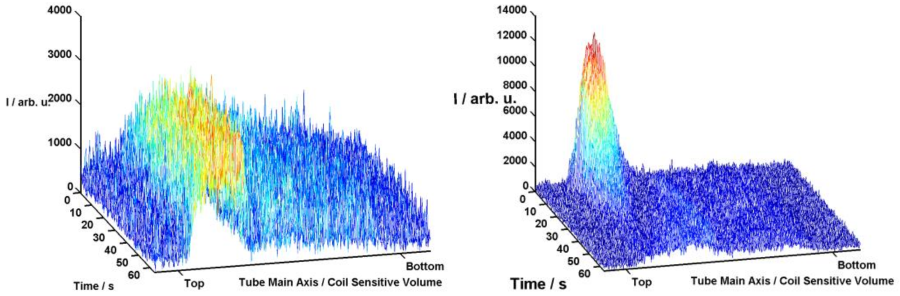

Figure S68. 1D projection of the substrate signal after shaking the sample for 10 seconds as a function of time. Left: thermal equilibrium conditions. Right: SABRE hyperpolarized.

2.5 SABRE hyperpolarization of pyrazine in $CDCl_3/D_2O$ mixtures in the presence of NaCl: effect of temperature

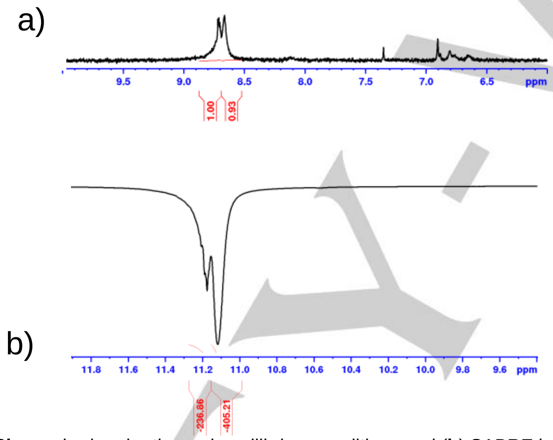

Figure S69. (**a**) ^{1}H NMR spectra of sample **3h** acquired under thermal equilibrium conditions and (**b**) SABRE hyperpolarized trace at 310 K.

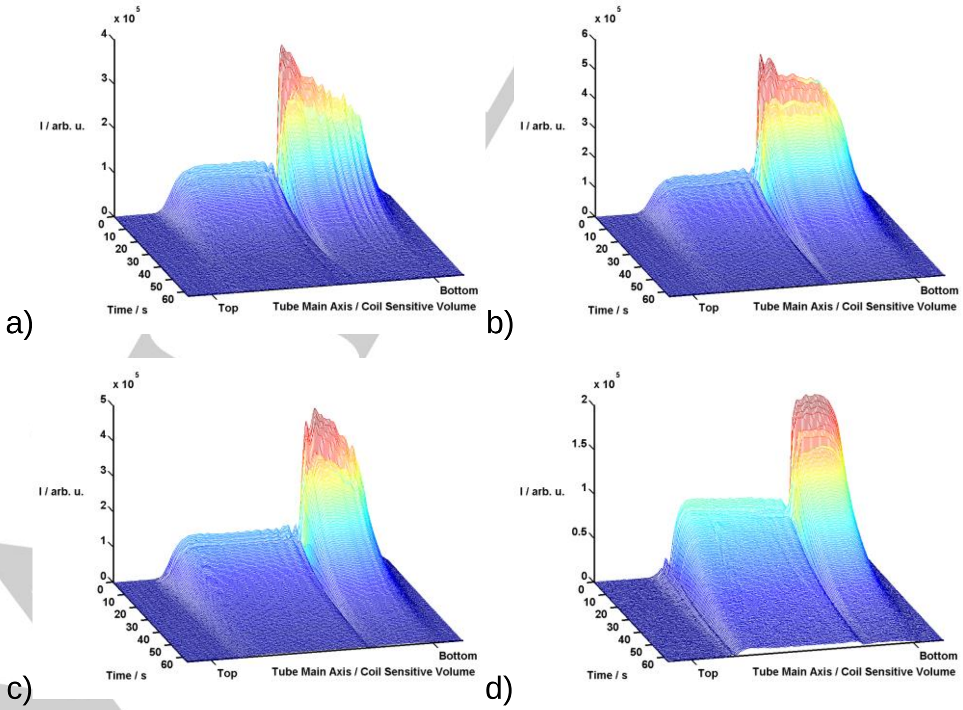

Figure S70. 1D projection of the substrate signal profile after shaking the sample for 10 seconds as a function of time at: a) 268 K, b) 298 K, c) 310 K and d) 320 K.

3. 2D MRI results

3.1 2D MRI of SABRE hyperpolarized pyrazine in $CDCl_3/D_2O$ mixtures in the presence of NaCl.

Prior to 2D data acquisition, 1D projections of the signal derived from the hyperpolarized protons of pyrazine have been acquired on samples prepared with 1-fold excess of ligand, a 17-fold excess of ligand and a 17-fold excess of ligand in the presence of 50 µl of EtOD. These results are presented in Figures S71 - S75.

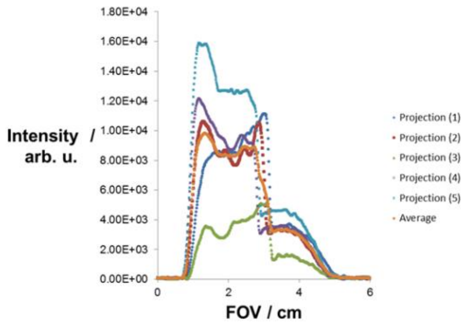

Figure S71. 1D MRI projections of the ^{1}H SABRE hyperpolarized signal of pyrazine (sample **2i**).

These data show that in the absence of EtOD the pyrazine is uniformly distributed in both $CDCl_3$ and D_2O, with average ratios of ~8:3 for the sample containing 1 fold excess and 5:1.5 for the sample containing 17-fold excess. EtOD addition therefore promotes the transition of the ligand dissolved in $CDCl_3$ towards the interface with D_2O (Figure S72, right).

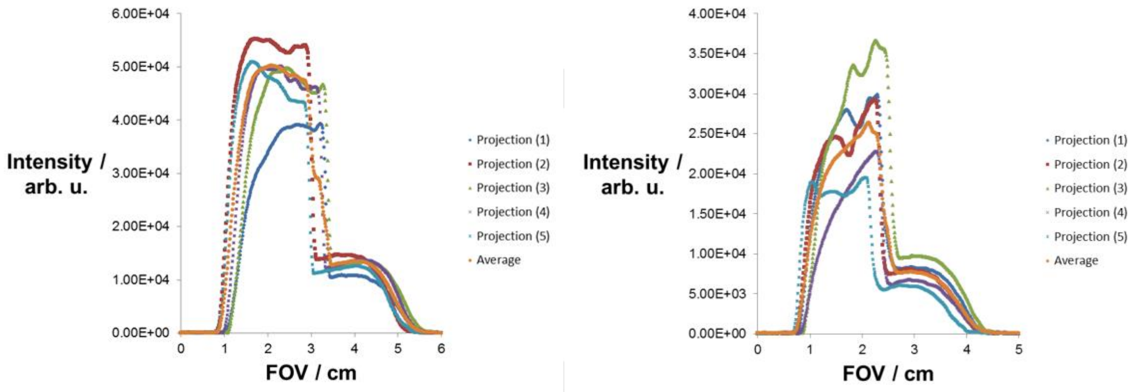

Figure S72. 1D MRI projections of the ^{1}H SABRE hyperpolarized signal of pyrazine. The samples have been prepared using 17-fold excess of ligand, left: sample **2g**, right: sample **2h**.

2D images of the samples have been acquired using a RARE protocol with centric *k*-space sampling in thermal equilibrium conditions and after 10 s of shaking in the stray field of the magnet in the presence of p-H_2. The results are depicted in Figures S73-S75.

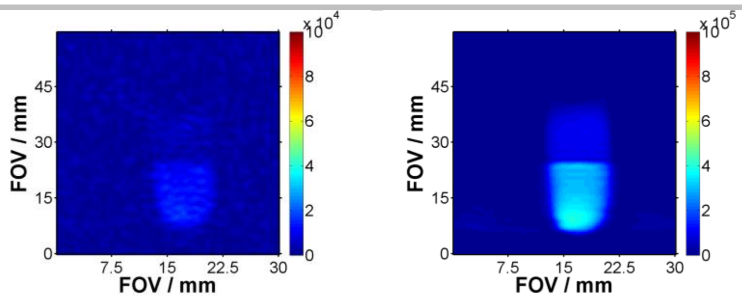

Figure S73. 2D RARE images of the ^{1}H SABRE hyperpolarized signal of pyrazine (sample **2i**). Left: thermal equilibrium, right: hyperpolarized.

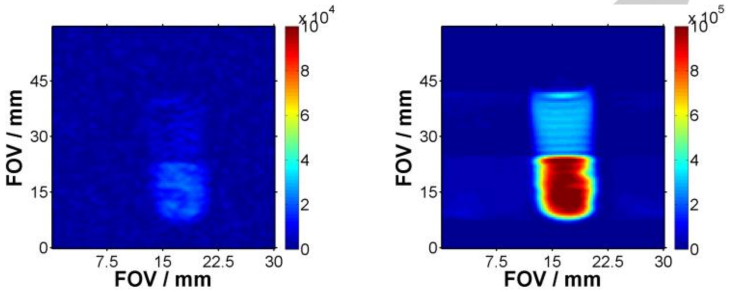

Figure S74. 2D RARE images of the ^{1}H SABRE hyperpolarized signal of pyrazine (sample **2g**). Left: thermal equilibrium, right: hyperpolarized.

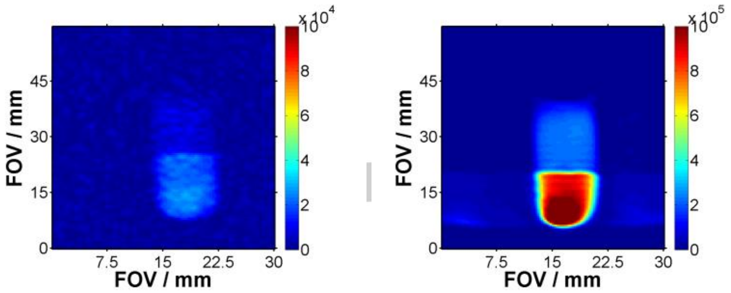

Figure S75. 2D RARE images of the ^{1}H SABRE hyperpolarized signal of pyrazine (sample **2h**). Left: thermal equilibrium, right: hyperpolarized.

In order to assess the evolution of the hyperpolarized signal as a function of time in both phases, gradient echo images acquired using a low flip angle pulse for excitation have been acquired each 0.8 s after the hyperpolarization step. The results, presented in Figure S76, show that, in a first instance, a more intense signal is obtained in the $CDCl_3$ phase. After longer acquisition times, the signal intensity in the D_2O phase becomes comparable with that of the signal in the $CDCl_3$ phase, demonstrating the continuous diffusion of hyperpolarized pyrazine in water.

It is worth noting that, in the sample containing EtOD, the highest signal intensity is obtained at the interface, as previously shown by the 1D projections. This reflects the possibility of using SABRE in the study of interface processes in multi-phase systems.

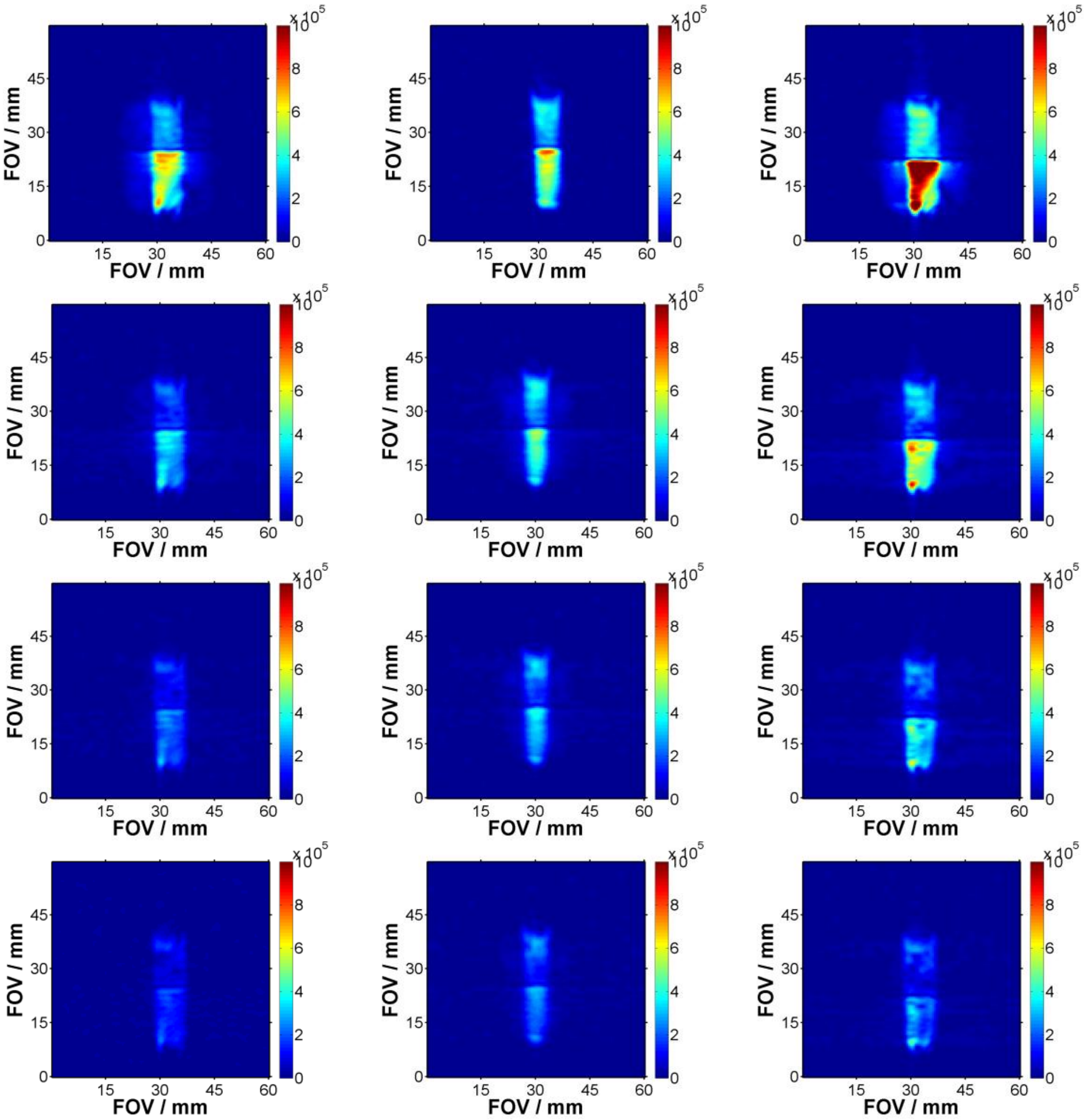

Figure S76. 2D MRI images of the ^1H SABRE hyperpolarized signal of pyrazine as a function of time. Images have been acquired (from top to bottom) after 0.8, 1.6, 2.4 and 3.2 s from inserting the sample into the imaging system. Left: sample **2i**, middle: sample **2g**, right: sample **2h**.

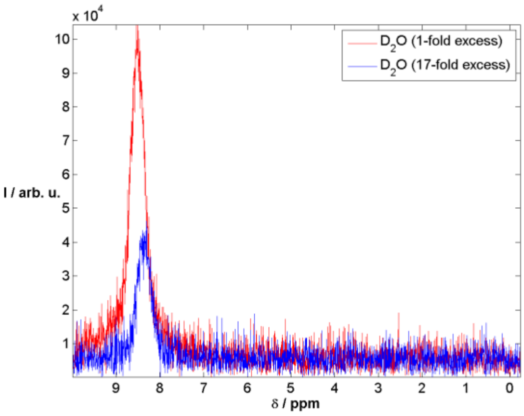

Figure S77. Single voxel response (SVS) of pyrazine in water as a function of substrate excess.

3.2 2D MRI of SABRE hyperpolarized pyrazine in $CDCl_3/D_2O$ mixtures in the presence of other salts.

Prior to 2D data acquisition, 1D projections of the signal derived from the hyperpolarized protons of pyrazine have been acquired on samples **3f** and **3g**, prepared with 1-fold excess of ligand and 3.3 mg $NH_4^+CH_3COO^-$, and 17-fold excess of ligand and 3.3 mg $NH_4^+CH_3COO^-$ + 1mg NaCl respectively. The results are presented in Figure S78.

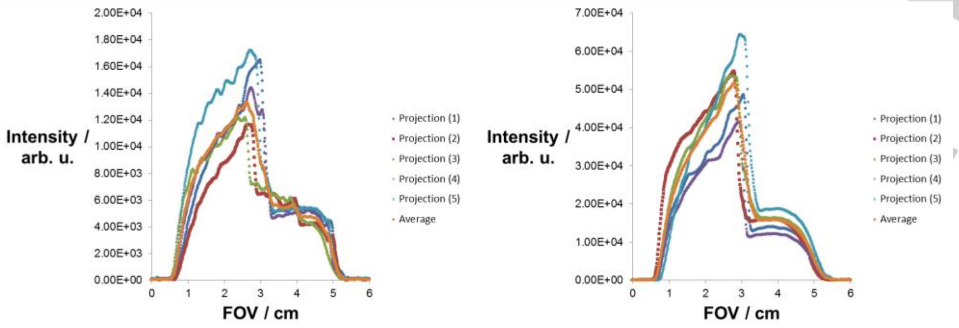

Figure S78. 1D MRI projections of the ^{1}H SABRE hyperpolarized signal of pyrazine: left: sample **3f** 1-fold excess), 3.3 mg $NH_4^+CH_3COO^-$, right: **3g** 17-fold excess, 3.3 mg $NH_4^+CH_3COO^-$, 1 mg NaCl.

2D images of the samples have been acquired using a RARE protocol with centric *k*-space sampling in thermal equilibrium conditions and after 10 s of shaking in the stray field of the magnet in the presence of *p*-H_2. The results are depicted in Figure S79 for the sample prepared with a 1-fold excess of pyrazine and Figure S80 for the sample prepared with 17-fold excess of pyrazine.

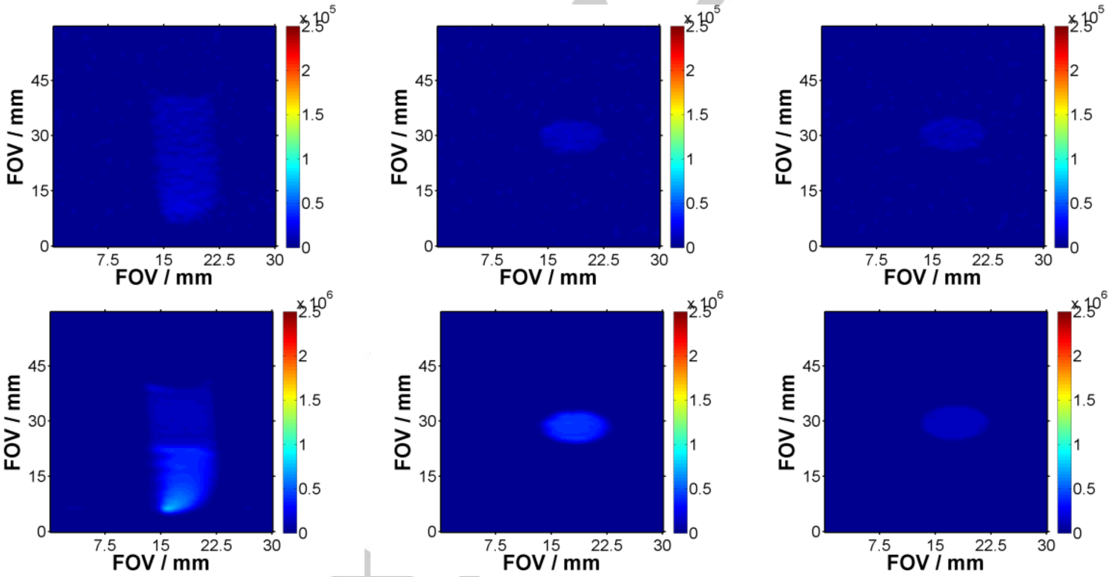

Figure S79. 2D MRI images the ^{1}H signal of pyrazine (1-fold excess + 3.3 mg $NH_4^+CH_3COO^-$ (sample **3f**)). From left to right: sagittal image of the whole tube, axial projection (5 mm slice) through the part of the tube containing $CDCl_3$, axial projection (5 mm slice) through the part of the tube containing D_2O. Top: thermal equilibrium conditions, bottom: SABRE hyperpolarized.

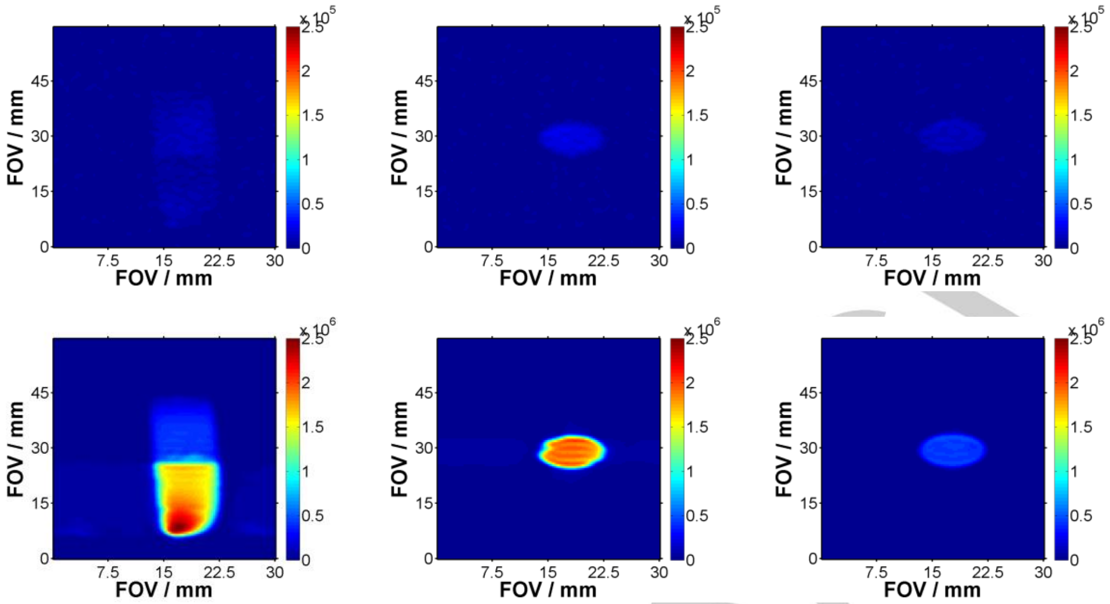

Figure S80. 2D MRI images the ^{1}H signal of pyrazine (17-fold excess + 3.3 mg $NH_4^+CH_3COO^-$ + 1 mg NaCl, sample **3g**). From left to right: sagittal image of the whole tube, axial projection (5 mm slice) through the part of the tube containing $CDCl_3$, axial projection (5 mm slice) through the part of the tube containing D_2O. Top: thermal equilibrium conditions, bottom: SABRE hyperpolarized.

3.3 2D MRI of SABRE hyperpolarized pyridazine in $CDCl_3/D_2O$ mixtures in the presence of NaCl.

In order to demonstrate that the CASH-SABRE approach, exemplified so far mainly for pyrazine, can be extended to other substrates, we have also tested the possibility of MRI detection of hyperpolarized pyridazine, which, as previously shown, tends to successfully concentrate in the aqueous phase, thus making this substrate a good candidate for *in vivo* MRI applications.

2D images of a sample containing 7-fold excess pyridazine have been acquired using the RARE protocol described previously. The results, presented in Figure S81, show that, after phase separation, excellent signal enhancement can be detected in the aqueous phase (top of the tube, see sagittal image) and almost no hyperpolarized pyridazine response is present in the organic phase.

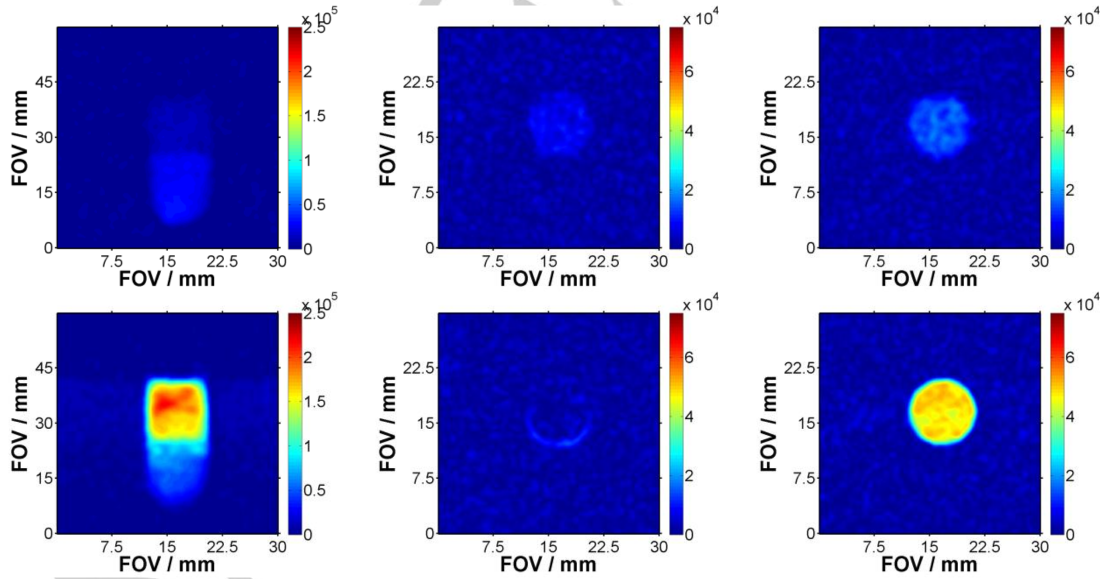

Figure S81. 2D MRI images the ^{1}H signal of pyradizine (7-fold excess + 20 mg NaCl). From left to right: sagittal image of the whole tube, axial projection through the part of the tube containing $CDCl_3$, axial projection through the part of the tube containing D_2O. Top: thermal equilibrium conditions, bottom: SABRE hyperpolarized. We note that the images presented in Figure S81, Figure S64 and Figure S68 show that both 4,6-d_2-nicotinamide and pyradizine present signal in the aqueous phase where they are highly soluble.

4. Quality control by UV-Vis and transport between the phases

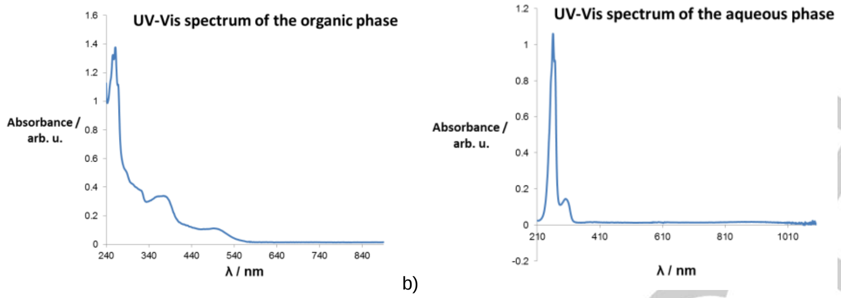

a) b)

Figure S82. (a) UV-visible spectrum of the chloroform layer after dilution (100 times). In this layer the catalyst is characterized by two absorption bands at λ_1 = 374 nm (ε = 6800 l.mol^{-1}.cm^{-1}) and λ_2 = 500 nm (ε = 2200 l.mol^{-1}.cm^{-1}) with pyrazine providing the λ_1 261 nm signal. (b) UV-visible spectrum of the D_2O layer after dilution of 10 times. In this layer, the pyrazine proved to be the main product.

A UV spectrum was recorded to monitor the amount of catalyst present in the aqueous phase 10 seconds after mixing. As detailed in Figure S83 no signal is seen which places an upper limit of 1.5 x 10^{-6} mol dm^{-3} on the iridium concentration based on [Ir(H)$_2$(IMes)(pz)$_3$]Cl. Further working using ICPMS is on-going to quantify this level more accurately. The sample used mimicked that of **2c**.

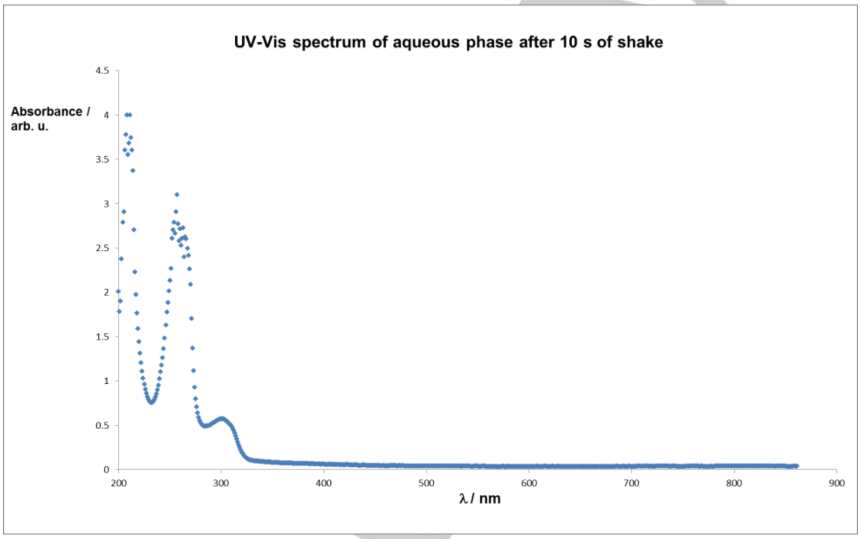

Figure S83. UV-visible response of the water phase that results 10 seconds after phase separation has started.

Relative rates of **pz** transfer from the aqueous phase into chloroform were assessed, with, and without NaCl, by UV monitoring as detailed in Figure S85. This involved layering a sample of H_2O containing pyrazine over an equivalent volume of $CHCl_3$ that contained no pyrazine. It appears that while the relative partioning of pz between these phases was unaffected by added NaCl (within the error of a control measurement) but the presence of NaCl was found to increase the rate of pz transfer between the phases.

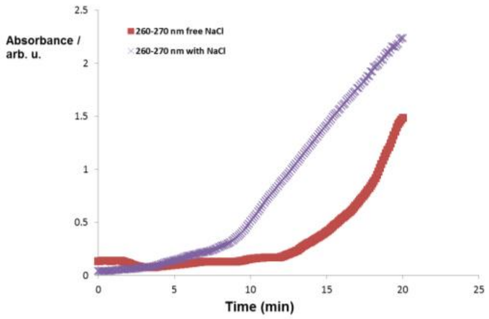

Figure S85. UV-visible response of pz in the chloroform phase as a function of time after the shake.

References

1. P. J. Rayner, M. J. Burns, A. M. Olaru, P. Norcott, M. Fekete, G. G. R. Green, L. A. R. Highton, R. E. Mewis, S. B. Duckett, *Proc. Natl. Acad. Sci.* **2017**, *114*, E3188-E3194.

WILEY-VCH
